# Functional magnetic resonance imaging in neuropathic pain: a bibliometric analysis incorporating machine learning

**DOI:** 10.3389/fnins.2026.1963495

**Published:** 2026-09-30

**Authors:** Ning Sun, Yu-Xin Zhu, Yi-Dan Cui, Shi-Qi Lu, Tian-Ran Gu, Ao Li, Jing-Zhi Wang, Yan Zou, Zhen Zhong, Hai-Peng Huang

**Affiliations:** 1 College of Acupuncture and Massage, Changchun University of Chinese Medicine, Changchun, Jilin, China; 2 Department of Spleen and Gastric Diseases, Liaoyuan Hospital of Traditional Chinese Medicine, Liaoyuan, Jilin, China

**Keywords:** bibliometrics, functional magnetic resonance imaging, machine learning, neuroimaging, neuropathic pain

## Abstract

**Background:**

Neuropathic pain (NP) affects 6.9%–10% of the global population. Functional magnetic resonance imaging (fMRI) has become central to NP investigation, yet few studies have systematically mapped this literature. We used bibliometric and machine-learning methods to chart the fMRI-NP research landscape.

**Methods:**

We retrieved 1,866 fMRI-NP articles (2005–2025) from Web of Science, PubMed, and Scopus. Following PRISMA guidelines, we analyzed publication trends, citation metrics, and collaborative networks with CiteSpace and VOSviewer, complemented by exploratory topic modeling based on term frequency–inverse document frequency (TF-IDF) and non-negative matrix factorization (NMF), and by community detection combining GraphSAGE node embeddings with density-based spatial clustering (DBSCAN).

**Results:**

The corpus amassed 94,160 citations, with an average of 50.46 per article and a corpus h-index of 139. Annual output rose from 35 articles in 2005 to 156 in 2025, following a power-law trend (R^2^ = 0.862). The United States and China were the most productive countries. Harvard Medical School and Massachusetts General Hospital were the most prolific institutions. NeuroImage and Pain were the most productive journals, and NeuroImage, the Journal of Neuroscience, and Pain were the most frequently co-cited. Text-mining of titles and abstracts indicated that stimulus-evoked activation, brain-connectivity/default-mode, and post-herpetic/regional themes were the most prevalent, with the limbic and sensorimotor systems the most frequently mentioned pathways.

**Conclusion:**

This study provides a comprehensive bibliometric mapping of the fMRI-NP research landscape, characterizing its growth trajectory, the most productive countries, institutions and journals, the most co-cited references, and the recurrent themes in the literature. The mapping indicates increasing attention to brain connectivity, multimodal imaging, and computational text analysis. These bibliometric patterns delineate active and emerging areas of research activity and may help orient future literature-based inquiry and collaboration in the field.

## 1 Introduction

Neuropathic pain (NP) affects 6.9%–10% of the global population (van Hecke et al., 2014), and its prevalence continues to rise (Colloca et al., 2017). This condition places a considerable burden on healthcare systems worldwide (Liu et al., 2025). According to the International Association for the Study of Pain (IASP), NP results from lesions or diseases of the somatosensory nervous system, triggered by primary injuries, dysfunctions, or transient disturbances in the peripheral or central nervous systems (Jensen et al., 2011; Raja et al., 2020). Typical symptoms include burning, tingling, paresthesia (“pins and needles”), and electric shock-like sensations (Kritikou et al., 2023). If left untreated, NP often leads to anxiety and depression (Vadalouca et al., 2021), together causing a profound decline in patients’ quality of life (Leoni et al., 2025).

Despite this, only 30%–40% of patients experience satisfactory relief with pharmacotherapy (Moisset et al., 2020), and treatment efficacy tends to wane over time (Finnerup et al., 2018). The development of targeted drugs remains a major challenge (Bouhassira and Attal, 2017; Rice et al., 2021), while conventional neuromodulation methods such as transcutaneous electrical nerve stimulation (TENS) show limited benefit (Gibson et al., 2017). Even spinal cord stimulation (SCS) requires stronger efficacy validation in randomized controlled trials (Katz et al., 2021). Functional magnetic resonance imaging (fMRI), introduced in the 1990s as a non-invasive neuroimaging technique, offers a research platform for addressing these challenges (Attal et al., 2023). fMRI provides non-invasive, whole-brain mapping of BOLD-related functional activity with relatively high spatial resolution (Glover, 2011; Logothetis, 2008). These features make fMRI an essential platform for advancing therapeutic research.

Although the use of fMRI in NP research has grown substantially in recent years (Buehlmann et al., 2018; Cao et al., 2017; Fiúza-Fernandes et al., 2025; Park et al., 2023), systematic mapping of this neuroimaging-specific literature is still lacking. Bibliometric analyses of neuropathic pain do exist: Leoni et al. (2025) comprehensively mapped the overall NP research landscape, and related analyses have covered adjacent areas of pain research. However, these works did not focus on functional neuroimaging, did not integrate the three major databases (Web of Science, PubMed, and Scopus) into a harmonized corpus, and did not incorporate machine-learning-based topic and network analyses. In this study, we map the structure and development of the fMRI-NP literature using well-established bibliometric tools (CiteSpace and VOSviewer), complemented by a transparent text-mining layer that summarizes the thematic and collaborative structure of the corpus. The aim is to characterize publication output, collaborative patterns, influential references, thematic trajectories, and emerging research directions within the field. The research question was defined using the PICOS framework. The population comprised peer-reviewed original research articles applying fMRI to NP. The intervention of interest was fMRI-based investigation of NP. Comparators were not applicable, as the review maps the evidence base rather than comparing interventions. The outcomes were the publication trends, collaborative networks, citation impact, and thematic structure of the literature. Eligible study designs were English-language peer-reviewed original articles.

Our methodology integrates bibliometric analysis with a suite of machine-learning and text-mining techniques to examine research trends in depth. CiteSpace and VOSviewer were applied to delineate the structural evolution and distribution patterns of scientific knowledge in this field, offering a quantitative approach that complements narrative review (Chen, 2006). In parallel, the titles, abstracts, and author keywords of the corpus were processed with established text-mining methods to summarize its thematic and collaborative structure (Kreimeyer et al., 2017). Research themes were extracted using term frequency–inverse document frequency (TF-IDF) weighting followed by non-negative matrix factorization (Lee and Seung, 1999) and projected for visualization with Uniform Manifold Approximation and Projection (McInnes et al., 2018). Country collaboration structure was characterized through neighbor-aggregated node embeddings in the GraphSAGE framework (Hamilton et al., 2017), with communities identified by density-based spatial clustering DBSCAN (Xu et al., 1999) and displayed using t-distributed stochastic neighbor embedding (van der Maaten and Hinton, 2008). Annual publication output and country-pair collaboration were further described with a random-forest model (Breiman, 2001) and an interpretable power-law fit under a fixed random seed to ensure reproducibility. By systematically triangulating these outputs across temporal publication trends, country and institutional contributions, collaboration networks, journal influence, co-citation clusters, and keyword dynamics, we trace conceptual shifts, delineate current research frontiers, and describe emerging directions, thereby providing an empirical basis for advancing fMRI applications in neuropathic pain research. Each component was selected for a specific descriptive aim: TF-IDF–NMF summarizes the thematic structure of titles and abstracts, embedding-based clustering characterizes collaboration communities, and the random-forest and link-scoring models describe temporal trends and collaborative patterns in an exploratory, hypothesis-generating manner.

## 2 Methods

### 2.1 Data sources and search strategies

All literature examined in this study was retrieved from the Science Citation Index Expanded (SCI-EXPANDED) within the Web of Science Core Collection (WoSCC), PubMed and Scopus. The search covered publications from 1 January 2005 to 31 December 2025, with retrieval performed on 31 May 2026. The specific search strategy for WoSCC is presented in Table 1. The other search strategies were given in Supplementary Table 1. Searches were limited to peer-reviewed, English-language journal articles. The three database-specific strategies use identical term blocks adapted to each platform’s syntax and are reported verbatim in Table 1 and Supplementary Table 1.

**TABLE 1:** Search strategies for WoSCC.

Rank	Search strategies
#1	TS = (“neuralgia*” OR “neurodynia*” OR (neuropath* AND pain*) OR (neurogenic AND pain*) OR (neuralgi* AND pain*) OR (nerve AND pain*) OR “painful polyneuropath*” OR “painful radiculopath*”)
#2	TS = (“fMRI” OR (“functional magnetic resonance imaging”) OR (“BOLD-fMRI”) OR (“Functional MRIs”) OR (“functional MR imaging”) OR (“resting-state fMRI”) OR (“functional MRI”) OR (“rs-fMRI”) OR (“Resting State Functional Magnetic Resonance Imaging”) OR (“resting state fMRI”))
#3	#1 AND #2

### 2.2 Eligibility criteria

In line with the PICOS framework defined above, the following eligibility criteria were applied. Peer-reviewed articles published in English were included, without geographical restrictions. Exclusion criteria were: conference abstracts, proceedings, letters, editorials, book chapters, preprints, retracted papers, notes, protocol, review, corrigenda, commentaries; duplicate publications; records lacking full text; and documents irrelevant to the research scope. The inclusion threshold was a database-retrieved fMRI study of neuropathic pain confirmed at full-text review. Records without an explicit fMRI term in the parsed title, abstract, or author-keyword fields were retained only when full-text screening confirmed that fMRI was used. All retrieved reports underwent full-text eligibility assessment, and no reports were excluded at this stage because they satisfied all predefined inclusion criteria. Because the search strategy was deliberately broad to capture the full spectrum of pain-related fMRI research, the corpus also includes studies of conditions in which neuropathic, nociplastic, or mixed pain mechanisms are debated, such as migraine, fibromyalgia, and irritable bowel syndrome (Treede et al., 2019; Fitzcharles et al., 2021). These records were retained when they satisfied the search and eligibility criteria, and their presence is quantified transparently in the keyword and topic analyses (Figures 6, 9, 10 and Table 2).

**TABLE 2:** Top 25 keywords.

Rank	Keyword	Frequency	First year	Peak year
1	Functional connectivity	214	2008	2022
2	Resting state	163	2008	2020
3	Chronic pain	127	2008	2025
4	Migraine	69	2010	2017
5	Insula	53	2005	2013
6	Default mode network	51	2008	2016
7	Empathy	45	2005	2013
8	Acupuncture	39	2005	2025
9	Fibromyalgia	39	2008	2016
10	Nociception	31	2006	2017
11	Trigeminal neuralgia	31	2012	2025
12	Amygdala	29	2006	2012
13	Brainstem	28	2005	2020
14	Anterior cingulate cortex	28	2005	2016
15	Chronic low back pain	27	2009	2025
16	Postherpetic neuralgia	27	2014	2018
17	Thalamus	26	2008	2022
18	Reho	25	2012	2024
19	Plasticity	24	2005	2015
20	Spinal cord injury	22	2006	2024
21	Irritable bowel syndrome	21	2006	2014
22	Effective connectivity	20	2012	2016
23	Hyperalgesia	19	2005	2008
24	Headache	19	2006	2017
25	Graph theory	19	2014	2021

### 2.3 Selection of sources of evidence

Before title and abstract screening, records were excluded according to the predefined publication-type and language criteria (non-English publications, data papers and retracted publications, conference papers and abstracts, editorial materials, book chapters and letters, preprints, and reviews). Two evaluators independently screened the titles and abstracts of all remaining records. Before the formal screening, the two evaluators were assigned a randomly selected sample of 50 records to conduct a preliminary screening test to ensure the reliability of the screening process. The final Cohen’s kappa value was 0.88, indicating high consistency, and no modifications were made to the eligibility criteria or the evaluators. During the formal independent screening, any disagreements were resolved by a third reviewer through team discussion. Deduplication was carried out in two stages: exact matching on the Digital Object Identifier (DOI), followed by fuzzy matching on normalized titles (lower-cased, with punctuation and redundant whitespace removed) combined with first-author surname and publication year. Metadata were then harmonized to reconcile heterogeneous formatting across sources: journal titles were mapped to a single canonical form, and country names were standardized (for example, “USA” and “United States,” or “Peoples R China” and “China,” were unified). Inconsistent author and institution names were consolidated by normalizing letter case and diacritics, reconciling abbreviated and full forms of author names, and merging institutional name variants and parent–subsidiary designations into a single label. A final manual check of the merged records for residual duplication and field completeness was performed before analysis. For institution-level analyses, affiliation strings were parsed from the address field and harmonized to top-level organizations: department-, school-, and centre-level units were attributed to their parent organization, and spelling and name variants were merged using a documented thesaurus (Supplementary Table 14). Institutions were credited by full counting, so that a publication listing several organizations contributes one credit to each. Dual affiliations (for example, Harvard Medical School together with Massachusetts General Hospital) therefore appear under both entities. Harvard Medical School and its affiliated teaching hospitals were retained as separate entities because they are independent organizations with distinct addresses in the source records. This follows standard full-counting practice and avoids suppressing the visibility of either entity.

### 2.4 Data charting and bibliometric indicators

Records were pre-processed using Python 3.10.11 and analyzed in CiteSpace 6.4.R1 and VOSviewer 1.6.20 for temporal, country, institutional, author, journal, reference, and keyword analyses. For the keyword timeline overlay analysis (Figure 10A), CiteSpace 7.0.1 was used with 1-year time slices from 2005 to 2025; nodes were selected per slice using the g-index with k = 2 (LRF = 2.5, L/N = 10, LBY = 5, e = 1.0), and the resulting network was pruned with the Pathfinder algorithm. In addition to publication volume and betweenness centrality, we report the following impact indicators: total citations and average citations per article derived from the Times-Cited field, as well as the h-index, which is defined as the largest number h such that h items have at least h citations. This index is utilized to summarize cumulative impact at the corpus, country, institution, author, journal, and co-cited-author levels. Betweenness centrality measures a node’s bridging position within the network, capturing how often it lies on the shortest paths connecting other nodes. Country and region attribution was derived from author affiliation addresses, and international collaboration was defined as co-authorship involving at least two different countries or regions. Citation counts were taken from the Times-Cited field of the Web of Science Core Collection records. The charted data items comprised bibliographic metadata (authors, institutional affiliations, countries/regions, and journals), citation fields (Times-Cited counts and reference lists), and text fields (titles, abstracts, and author keywords), together with the derived bibliometric indicators described above. Data charting was independently performed by two reviewers, and discrepancies were resolved by consensus.

### 2.5 Machine-learning workflow

Analyses were conducted in Python with a fixed random seed (42). Requirement package versions were listed in the Supplementary Table 2. The title, abstract, and author keywords were concatenated and converted to lowercase. For topic modeling, the core topic-defining terms (such as neuropathic pain, fMRI/functional magnetic resonance imaging, brain, etc.) were excluded. Research themes were extracted using term frequency–inverse document frequency (TF-IDF; min_df = 5, max_df = 0.5, max_features = 300; unigrams and bigrams) followed by non-negative matrix factorization (NMF; n_components = 6, max_iter = 200) (Lee and Seung, 1999) and projected with Uniform Manifold Approximation and Projection (UMAP; n_neighbors = 15, n_components = 5) (McInnes et al., 2018). NMF was chosen because its additive, parts-based decomposition yields directly interpretable document–topic weights for short bibliographic texts, and the six-topic solution was selected after comparing four- to eight-topic solutions for coherence and interpretability. Document-level method, conclusion, and pathway frequencies were obtained through whole-word matching against predefined dictionaries, whose full term lists are given in Supplementary Table 12. Temporal change points in the annual topic-share matrix were detected with the Pelt algorithm (radial basis function cost, penalty = 2) implemented in the ruptures library (Truong et al., 2020). Country collaboration communities were identified from GraphSAGE-style neighbor-aggregated node embeddings (Hamilton et al., 2017) using DBSCAN (Xu et al., 1999), with ε = 1.630 derived from the 75th percentile of the k-distance curve and min_samples = 2, and were visualized with t-SNE [perplexity = min(30, n−1)] (van der Maaten and Hinton, 2008); keyword communities were detected with the Louvain modularity-optimization algorithm (Blondel et al., 2008) under the same fixed seed. Embedding-based community detection was run on the 49 countries listed in Table 3, the subset of the 196-country full-period network retained after filtering by publication volume for stable embedding estimation. Dynamic network metrics used data-derived time windows. Annual publication counts and country-pair collaborations were modeled with random forest (Breiman, 2001) on polynomial year features (t, t^2^, t^3^): the random forest (300 trees, max_depth = 3) captures potential non-linearities without imposing a parametric functional form, and its restricted depth limits overfitting on the 21-point annual series. Random forest, an ensemble of bagged decision trees, provides a flexible yet regularized fit that is well-suited to capturing potential non-linear dynamics in the annual series of only 21 observations. An interpretable power-law model (y = a⋅t^b) was fitted as a mechanistic reference for cumulative growth. For exploratory collaboration link scoring, a random-forest classifier (300 trees, max_depth = 5, class_weight = balanced) used ten topology- and frequency-based features (Supplementary Table 11), and features were screened to avoid leakage of edge identity. All models were assessed through 5-fold cross-validation (stratified for the classifier) and reported as mean ± SD. These exploratory models are used for descriptive purposes and hypothesis generation rather than prediction.

**TABLE 3:** Latent country-collaboration communities (GraphSAGE + DBSCAN; countries treated as noise by DBSCAN are listed as unclassified).

Community	Size	Countries
1	29	Finland; Canada; Sweden; Germany; Switzerland; UK; Japan; Italy; Belgium; Brazil; Singapore; France; Netherlands; Denmark; Australia; Israel; South Korea; Hungary; Turkey; Spain; Austria; Colombia; Mexico; Czech Republic; New Zealand; Ireland; Pakistan; Portugal; India
2	3	Argentina; Lebanon; Russia
3	3	Malaysia; Serbia; Romania
4	2	South Africa; Luxembourg
Unclassified	12	USA; China; Greece; Norway; Poland; Chile; Iran; Cyprus; Slovakia; Egypt; Saudi Arabia; Croatia

## 3 Results

### 3.1 Selection of sources of evidence

The database searches yielded 3,964 records (Web of Science 958, PubMed 266, Scopus 2,740). Before screening, 202 duplicate records were removed and 1,116 records were excluded according to the predefined publication-type and language criteria (non-English studies 75; data papers and retracted publications three; conference papers and abstracts 73; editorial materials 63; book chapters and letters 76; preprints 59; reviews 767). After removal of duplicates and records excluded for predefined reasons, 2,646 records were screened by title and abstract. Of these, 780 records were excluded because they were not aligned with the research topic (*n* = 764), lacked abstracts, full texts, or keywords (*n* = 15), or were study protocols (*n* = 1). Subsequently, 1,866 reports were sought for retrieval, all of which were successfully retrieved and assessed for eligibility against the predefined criteria. Full-text screening confirmed the eligibility of all retrieved reports, and no reports were excluded at the full-text assessment stage, resulting in 1,866 sources of evidence included in the review (Figure 1). In line with the scoping-review design, critical appraisal of the included sources was not performed. The characteristics of the included corpus are summarized below and in Tables 1–4 and Supplementary Tables 4–14.

**FIGURE 1:**
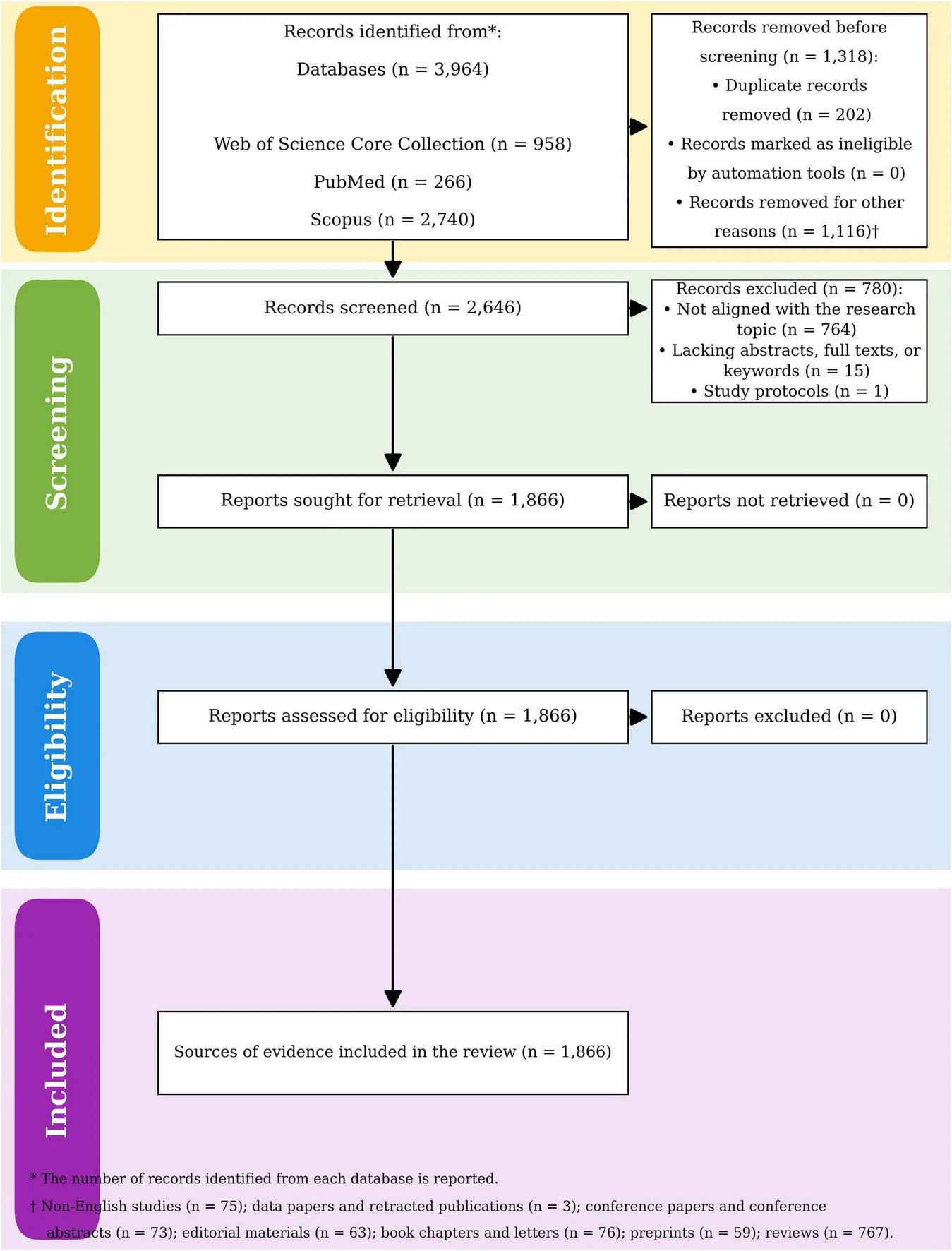
Flowchart of the study.

**TABLE 4:** Research themes of the most highly co-cited references.

References	Keyword	Direction	Method	Conclusion	Location
Apkarian et al., 2005	Positron-emission-tomography; laser-evoked-potentials; cerebral-blood-flow; anterior cingulate cortex; primary somatosensory cortex; irritable-bowel-syndrome; phantom-limb pain	Literature on how activity in different brain regions creates and regulates acute and chronic pain experiences	Hemodynamic (PET, fMRI), neuroelectrical (EEG, MEG) and neurochemical methods (MRS, receptor binding and neurotransmitter modulation)	Pain is strongly shaped by interactions between ascending and descending pathways	Primary and secondary somatosensory, insular, anterior cingulate, and prefrontal cortices (S1, S2, IC, ACC, PFC) and thalamus (Th)
Ong et al., 2018	Prefrontal cortex; pain; nociception; antinociception	Role of the prefrontal cortex in pain processing	Electrical Nerve-Stimulation;Resting State Connectivity	Evidence shows the PFC contributes to placebo analgesia	Prefrontal cortex (PFC)
Peyron et al., 2004	Somatosensory evoked-potentials; human anterior cingulate; post-stroke pain; thalamic pain; brain responses; insular cortex	Investigation of cerebral activity linked to allodynia in neuropathic pain	fMRI	Demonstrated the perceptual shift from innocuous to painful and poorly defined sensations	Primary (SI), secondary (SII) somatosensory cortices; insular regions; insular cortices; primary motor cortex (MI); parietal operculum (SII); mid-anterior cingulate cortex
Maihöfner et al., 2006	Reflex sympathetic dystrophy; induced secondary hyperalgesia; anterior cingulate cortex; mechanical hyperalgesia; cortical representation	Examination of cerebral activations underlying touch-evoked pain (dynamic-mechanical allodynia) in neuropathic pain patients	fMRI	Showed activation from sensory systems such as visual and vestibular cortices projecting into somatosensory-related brain areas	Contralateral S1 and motor cortex (M1), parietal association cortices (PA), bilateral S2, insula, frontal cortices, and both anterior and posterior parts of the cingulate cortex (aACC and pACC)
Alshelh et al., 2016	Astrocytes; infra-slow oscillations; orofacial pain; regional homogeneity; spinal trigeminal nucleus; thalamocortical rhythm	Association of neuropathic pain with altered infra-slow oscillations in the ascending pain pathway	Resting-state functional magnetic resonance imaging	Identified increased oscillatory activity in the ascending pain pathway, consistent with a self-sustaining thalamocortical dysrhythmia and persistent pain perception	The spinal trigeminal nucleus, somatosensory thalamus, thalamic reticular nucleus, and primary somatosensory cortex
Li et al., 2020	Postherpetic neuralgia; pain; thalamus; periaqueductal gray matter (PAG); magnetic resonance imaging (MRI); anxiety; depression	Deficits in ascending and descending pain modulation in PHN patients	Psychophysics with structural and functional magnetic resonance imaging (MRI)	Demonstrated pathway deficits closely linked to pain intensity and emotional comorbidities in PHN	Thalamus and periaqueductal gray matter (PAG)

### 3.2 Analysis of annual publications

Figure 2A illustrates three phases in publication trends. In Phase 1 (2005–2010), annual output fluctuated but showed a general upward trend, reaching an initial peak of 56 publications in 2009 before dropping to 45 in 2010. Phase 2 (2011–2019) displayed relatively minor variation with steady growth. Phase 3 (2020–2025) showed consistent progression, highlighted by a sharp rise to 156 publications in 2025.

**FIGURE 2:**
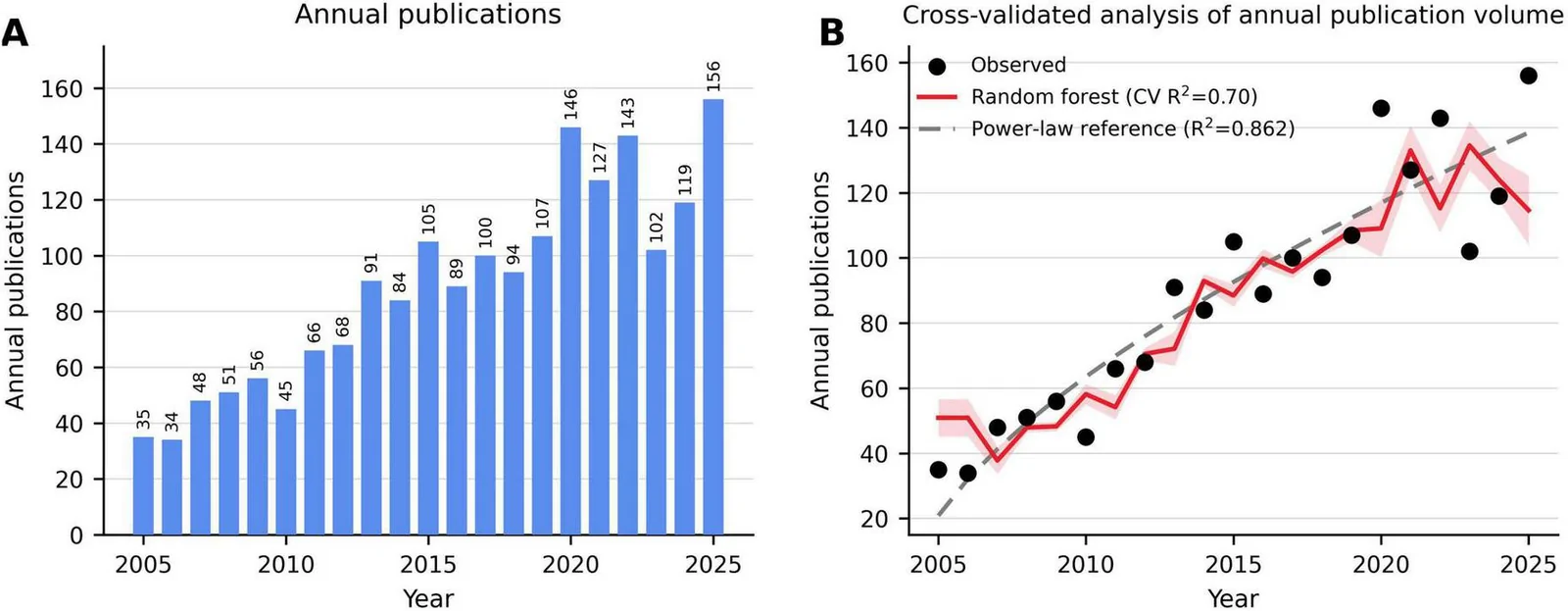
Analysis of annual publications. **(A)** Annual publications. **(B)** Cross-validated analysis of annual publication volume: points denote observed annual counts; the solid curve denotes out-of-fold predictions from 5-fold cross-validation for the random forest; the dashed curve denotes the fitted power-law reference (R^2^ = 0.862); the shaded band denotes ±1 SD across folds.

Annual output increased from 35 publications in 2005 to 156 in 2025, yielding a mean of 88.9 publications per year and an overall increase of 345.7%. To describe this growth trajectory more precisely, we applied a random-forest regression model (Figure 2B). Through 5-fold cross-validation, the random forest model achieved a fit of R^2^ = 0.7012 ± 0.1566 (MSE = 315.28, MAE = 13.84). Figure 2B displays the out-of-fold (cross-validated) predictions of the random forest model. An interpretable power-law model fit the series well (R^2^ = 0.862, an in-sample descriptive fit, in contrast to the out-of-fold cross-validated regression estimate above). Because random k-fold validation on a 21-point annual series is subject to temporal leakage, these regression results are interpreted as descriptive summaries of curve shape.

### 3.3 Analysis of national cooperative networks

CiteSpace was used to visualize collaborations among countries and regions (Figure 3A). The meaning of node size, annual rings, link color and thickness, and centrality rings in Figure 3A is described in the figure legend. Betweenness centrality values greater than 0.1 are considered significant. Nodes with high centrality are highlighted by purple rings, with thicker rings denoting stronger centrality.

**FIGURE 3:**
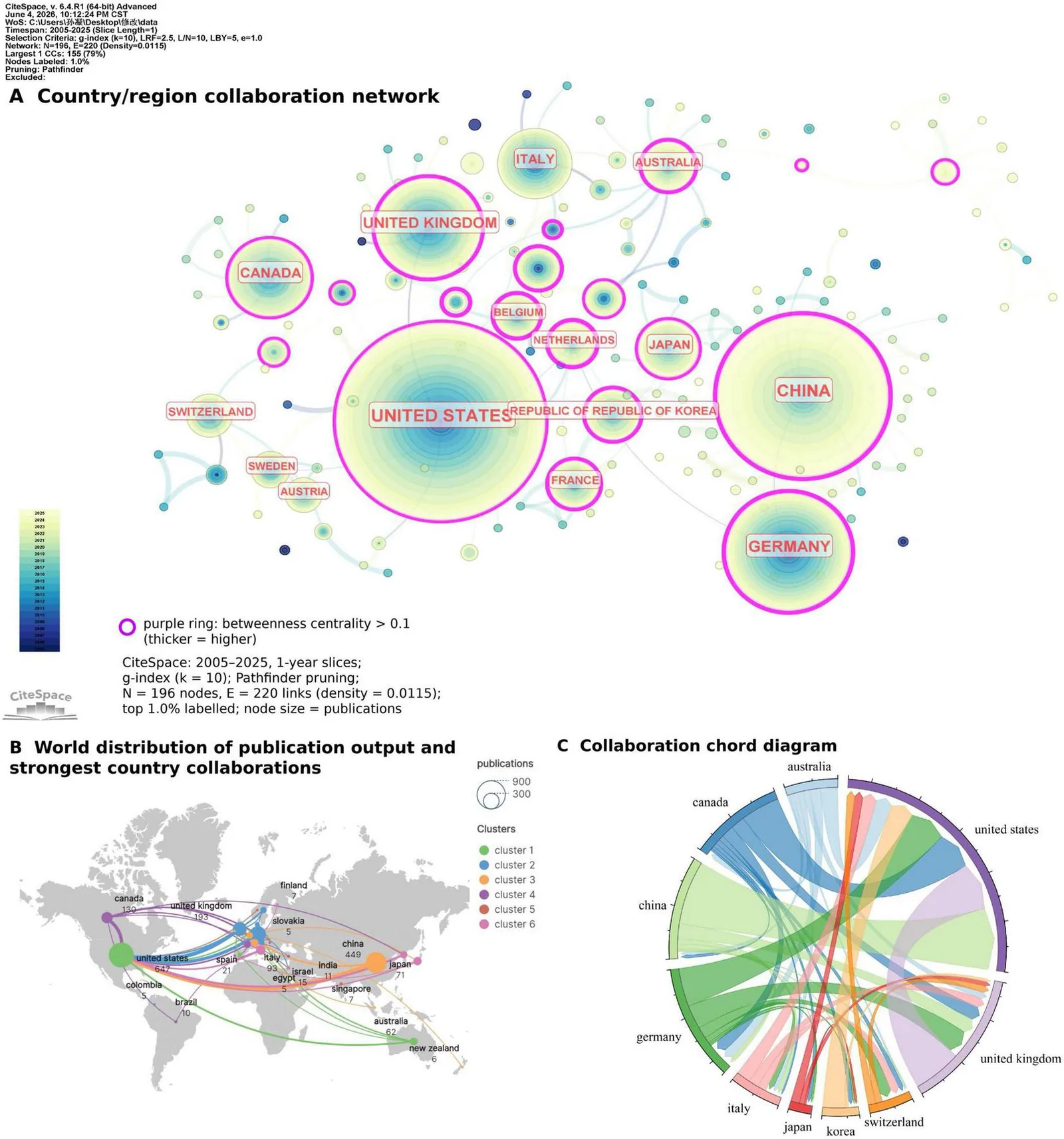
Analysis of national cooperative networks. **(A)** National cooperative networks: node size denotes the publication count per country; concentric annual rings denote yearly output; link color denotes the year of first collaboration and link thickness the number of co-authored publications; purple rings mark nodes with betweenness centrality greater than 0.1, with thicker rings denoting stronger centrality. **(B)** World distribution of publication output and the strongest country collaborations: node color denotes the collaboration community (Table 3; gray denotes unassigned countries), node size denotes the number of publications per country/region, connecting lines denote the strongest country pairs with line width proportional to the number of co-authored publications, and only the most productive countries are labeled. **(C)** Chord diagram of the collaboration among the ten most connected countries; ribbon width is proportional to the number of co-authored publications between each country pair.

The network contained 196 nodes and 220 links (Figure 3B). The United States led with 647 publications, followed by China (449), Germany (262), the United Kingdom (193), and Canada (130). These five nations each produced at least 100 papers. New Zealand showed the highest centrality (0.64), followed by Netherlands (0.59), Germany (0.52), China (0.51), and Belgium (0.48) (Supplementary Table 3). Given the low output of some high-centrality countries (for example, New Zealand: h-index 3), these centrality values are sensitive to single publications and should be interpreted with caution.

The US maintained extensive collaborations with all of the top-10 publishing countries, particularly China. China worked mainly with the US but had limited cooperation with other leading nations. Other countries displayed relatively balanced collaboration structures (Figure 3C).

Embedding-based community detection, utilizing GraphSAGE-style neighbor aggregation followed by DBSCAN (ε = 1.630), identified four latent communities among the countries (Table 3 and Supplementary Figure 1A). A further twelve countries were treated as noise by DBSCAN and are therefore listed as unclassified in Table 3. In the most recent time window, the collaboration network included 45 countries and 163 links, resulting in a density of 0.165 and a mean clustering coefficient of 0.605 (Supplementary Figure 1B). An exploratory random-forest link-scoring model, assessed through five-fold stratified cross-validation, distinguished between existing and absent collaborations, achieving an ROC-AUC of 0.921 ± 0.017 (positives 216/1176). Because the random edge split allows the same country to appear in both training and test edges, this ROC-AUC likely overestimates out-of-time performance. The high-scoring unlinked country pairs are therefore presented as exploratory hypotheses for future collaboration rather than as validated predictions (Supplementary Figure 1C). The ten link-scoring features are listed in Supplementary Table 11.

### 3.4 Network analysis of institutional collaboration

CiteSpace was used to map institutional collaboration networks within scholarly exchanges. A collaboration was defined as the co-occurrence of two institutional affiliations within a single publication. Node size reflects publication volume, with larger nodes indicating higher output. The citing half-life, a standard bibliometric indicator provided by the platform, was used to describe the obsolescence rate of an institution’s cited literature.

The institutional collaboration network is shown in Figure 4A. As summarized in Supplementary Table 4, the most productive institutions were Harvard Medical School (132 articles), Massachusetts General Hospital (110), the University of Oxford (58), University Medical Center Hamburg-Eppendorf (51), Northwestern University (40), and the University of Toronto (40). By citation indicators, the University of Michigan (h-index 32; 177.7 mean citations per article) and Northwestern University (h-index 32; 163.2 mean citations) showed the highest mean citation rates, whereas Harvard Medical School and Massachusetts General Hospital (h-index 58 each) accumulated the largest total citation counts. The most prolific institutions were predominantly based in the United States, and most collaborations occurred between universities and their affiliated hospitals and within national borders. The relatively low network density indicated limited international and inter-institutional cooperation. These counts describe the publication volume and citation frequency of each institution’s output.

**FIGURE 4:**
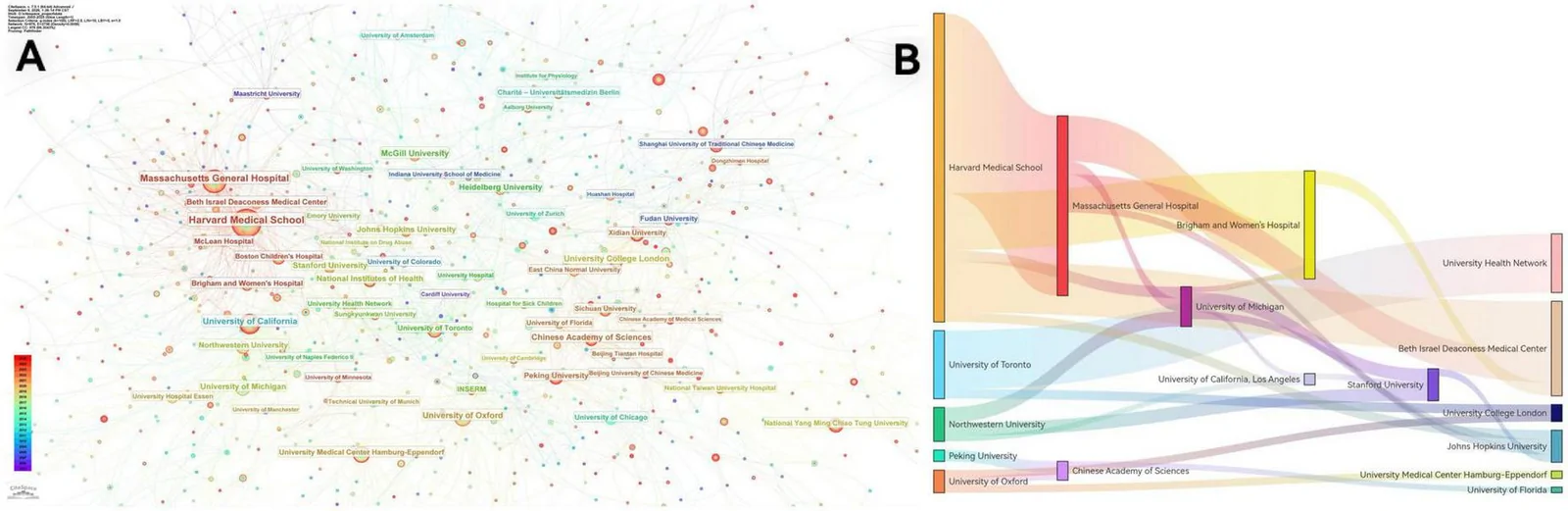
Network analysis of institutional collaboration. **(A)** Institutional cooperative networks. **(B)** Sankey diagram of the strongest collaboration links among the 20 most productive institutions: bar height denotes the number of co-authored publications of an institution within the displayed network, and band width is proportional to the number of co-authored publications between two institutions; only links representing at least three co-authored publications are shown, and bands run from the more to the less productive institution for display purposes only. Institutions were counted after affiliation harmonization (Supplementary Table 14).

Figure 4B illustrates the strongest collaboration links among the 20 most productive institutions as a Sankey diagram, in which band width is proportional to the number of co-authored publications and only links representing at least three co-authored publications are shown (17 of the 20 institutions met this criterion). The left-to-right direction runs from the more to the less productive institution for display purposes only. The strongest tie linked Harvard Medical School and Massachusetts General Hospital (95 co-authored publications), followed by the University of Toronto with the University Health Network (31), Harvard Medical School with Brigham and Women’s Hospital (30), and Harvard Medical School with Beth Israel Deaconess Medical Center (27). Within the United States, Harvard Medical School, Massachusetts General Hospital, Brigham and Women’s Hospital, and Beth Israel Deaconess Medical Center formed a densely interconnected collaboration cluster, and the University of Michigan, Northwestern University, and Stanford University formed another tightly connected group. The University of Toronto in Canada collaborated most strongly with its teaching hospitals within the University Health Network. In China, Peking University and the Chinese Academy of Sciences collaborated closely. Overall, universities tended to partner preferentially with their affiliated institutions, with collaborations largely occurring within the same country.

### 3.5 Analysis of the authors and co-cited authors

The co-authorship network map generated by VOSviewer (Figure 5A) depicts nodes representing individual authors, where node size corresponds to publication volume. Links between nodes indicate collaborations, with line thickness representing the strength of cooperation, quantified as the number of co-authored publications shared by two authors (the link-strength indicator in VOSviewer). Burst detection identified authors who showed sudden increases in productivity during specific periods.

**FIGURE 5:**
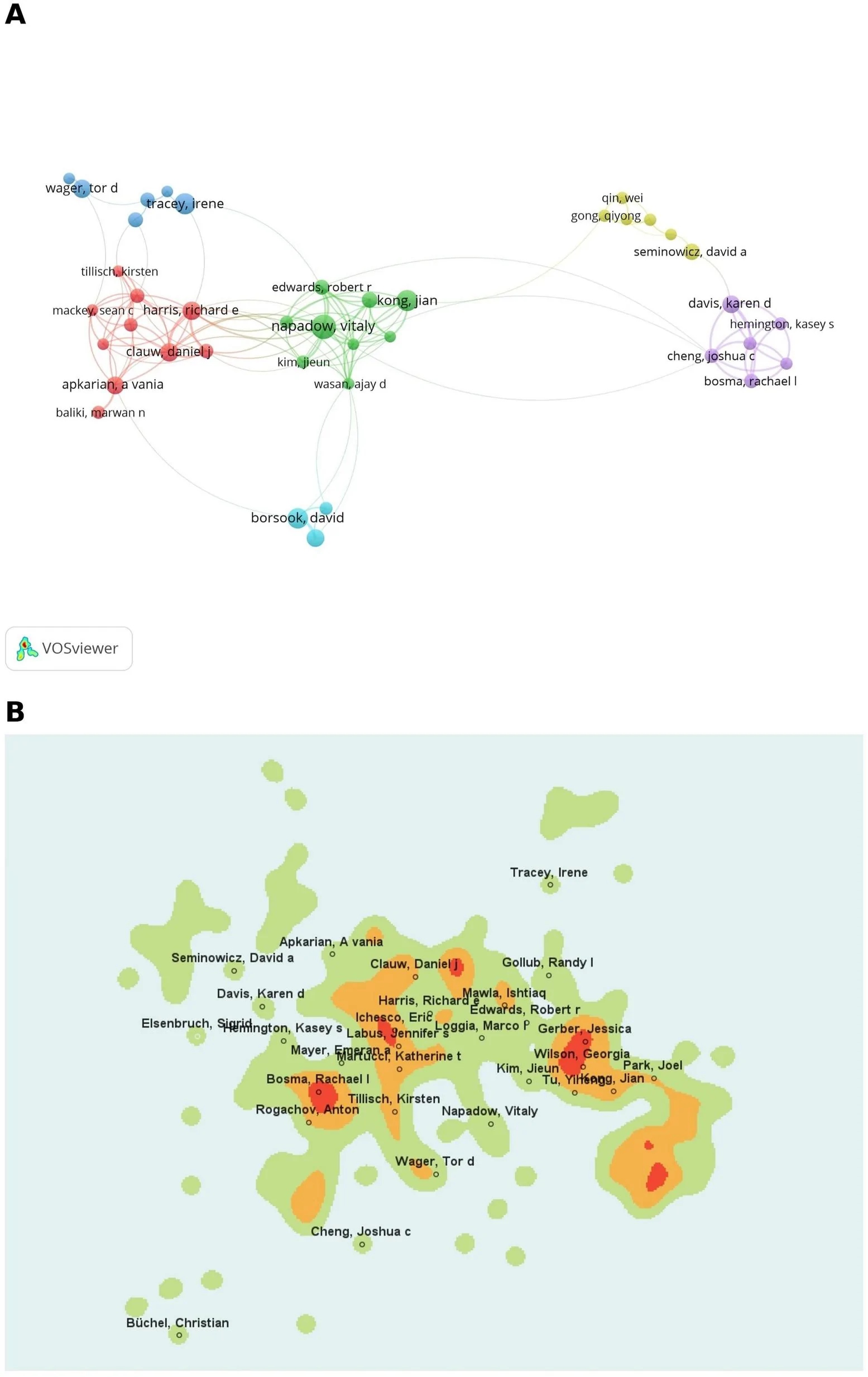
Analysis of the authors and co-cited authors. **(A)** Map of the authors: node size denotes publication volume and link thickness the number of co-authored publications. **(B)** Heatmap of co-authorship: color intensity denotes co-authorship frequency.

The most prolific authors were Napadow, Vitaly (*n* = 41), Tracey, Irene (*n* = 31), and Kong, Jian (*n* = 31), each contributing more than 30 publications (Supplementary Table 5). VOSviewer identified six collaboration clusters with stable cooperative relationships. Three research teams were particularly prominent: the group led by Napadow et al. (2023) focusing on fMRI applications across neuropathic pain conditions (Bolender et al., 202); Henderson’s et al., 2021 team investigating chronic pain mechanisms in the dorsolateral prefrontal cortex; and Gu Lili’s program concentrating on postherpetic neuralgia (Li et al., 2025). VOSviewer heatmaps (Figure 5B) visualized co-authorship intensity through color gradients, with darker shades reflecting greater co-authorship frequency. These team profiles are summarized from the titles and keywords of the corresponding corpus publications.

The Sigma index (Σ), a composite metric in CiteSpace, quantifies node novelty by combining structural importance (centrality) with temporal significance (burst). It is calculated as Sigma = (centrality+1)^burstness (Chen et al., 2010), highlighting nodes that combine high betweenness centrality with strong citation bursts.

Co-citation analysis identified the most frequently co-cited authors: Tracey I (464 co-citations), Davis KD (354), Apkarian AV (330), and Borsook D (289), followed by Raichle ME (256) and Wager TD (254) (Supplementary Table 6). These counts indicate how often an author’s work is cited together with that of others in the corpus and identify shared intellectual reference points. They identify the field’s shared intellectual anchors, and several of the most co-cited authors are foundational contributors whose work is widely referenced.

Python-based text mining of titles, abstracts, and keywords employed exact-match algorithms to define research domains (Supplementary Table 7). The full term lists of the underlying dictionaries are given in Supplementary Table 12. Most authors concentrated on pain mechanisms and brain networks, especially chronic pain and neuroplasticity. fMRI was the dominant imaging modality, while functional connectivity analysis was the primary methodological approach. Central findings concerned pain-related brain regions, alterations in functional connectivity, and treatment response. The limbic system and sensorimotor network were the most frequently investigated neural pathways, followed by the attention network and default mode network. Distinct author profiles emerged in terms of thematic focus, methodological choices, and reported conclusions.

Unsupervised topic modeling of the complete corpus (TF-IDF + NMF; Figure 6A) identified six themes: stimulus-evoked activation (47.7%), brain connectivity and default mode (21.3%), post-herpetic/regional studies (13.2%), migraine (7.0%), spinal cord injury and rodent models (6.9%), and treatment/neuromodulation (4.0%). These percentages indicate how frequently each theme is represented in the corpus, providing a quantitative summary of the literature’s thematic structure.

**FIGURE 6:**
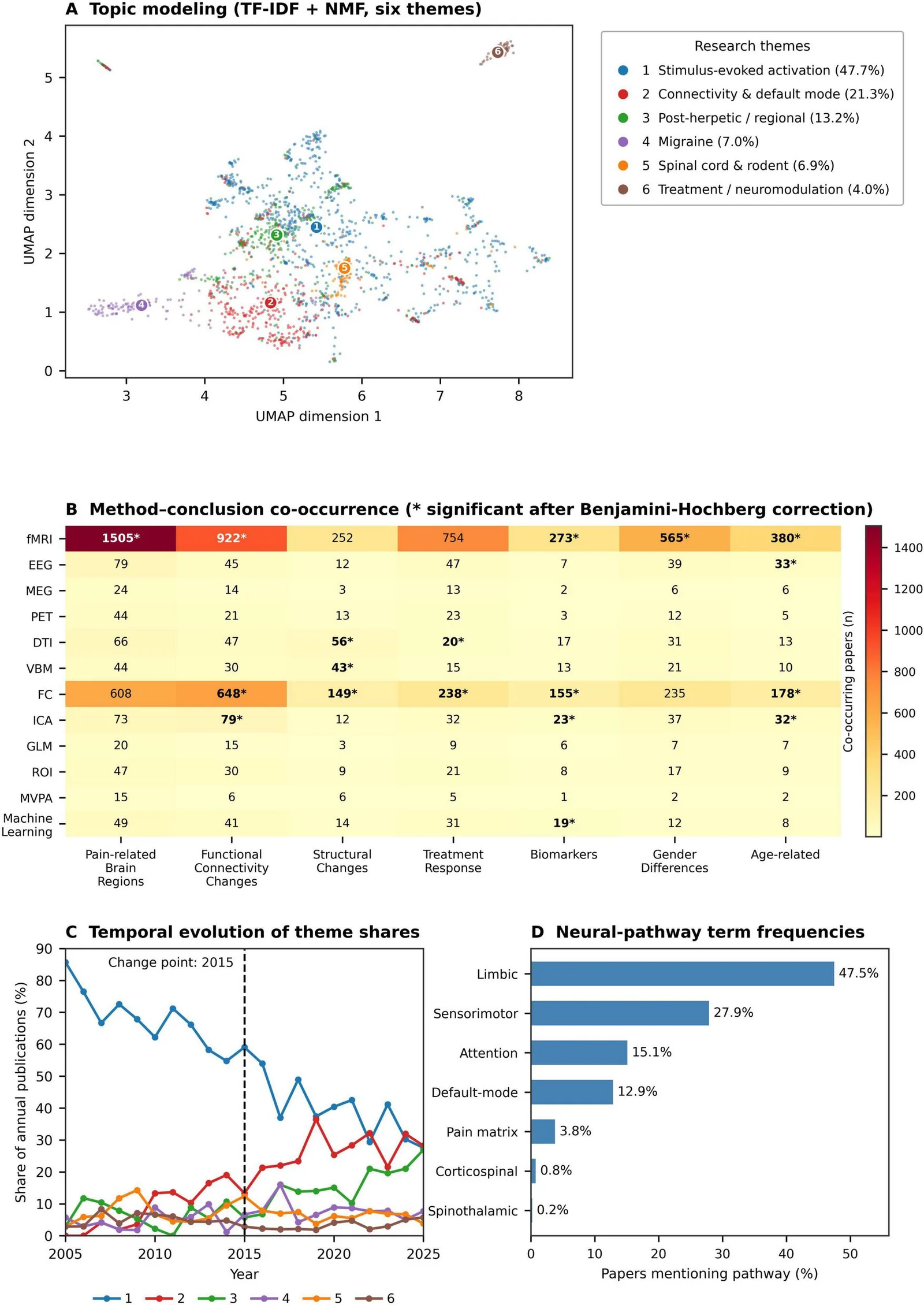
Text-mining analysis of research themes and methods. **(A)** Topic clustering of research themes. **(B)** Method–conclusion co-occurrence associations. **(C)** Temporal dynamics of research themes. **(D)** Neural-pathway mention frequencies. Topic colors are identical across panels **(A,C)**, and topic numbers are annotated directly in panel **(A)**. In panel **(B)**, rows and columns are dictionary categories, cell color and numbers denote method–conclusion co-occurrence counts, and asterisks mark associations that remain significant after Benjamini and Hochberg correction. In panel **(C)**, lines denote annual within-corpus shares of each theme, and the dashed vertical line marks the 2015 change point. In panel **(D)**, bars denote the percentage of papers mentioning each neural-pathway category.

Method and conclusion frequencies (Figure 6B) indicated that fMRI was the dominant modality, appearing in 86.4% of the studies. The remaining 13.6% of records (254 articles) entered the corpus through database indexing beyond the parsed title, abstract, and author-keyword fields (for example, controlled-vocabulary indexing) or used generic terms such as “neuroimaging,” so the 86.4% figure reflects whole-word dictionary coverage of the parsed text fields rather than off-scope inclusions. Non-fMRI modality terms such as EEG, MEG, PET, DTI, and VBM appear because many fMRI studies are multimodal or discuss alternative modalities, and their frequencies index co-mentions within the fMRI literature. Functional connectivity analysis was reported in 34.8% of the papers, while machine-learning methods were mentioned in 3.1%. The most frequently reported outcomes concerned pain-related brain regions (91.5%), functional connectivity changes (54.4%), and treatment response (47.3%). Following the Benjamini and Hochberg (1995) correction, 18 method–conclusion associations were statistically significant. These associations describe how often methods and reported outcomes co-occur in the literature, summarizing recurrent method–outcome patterns across the corpus.

Temporal analysis in Figure 6C revealed increasing trends over time for the connectivity and post-herpetic/regional themes, as well as for functional-connectivity and machine-learning method terms (from 21.5% and 0.6% of papers in 2010–2014 to 45.3% and 6.1% in 2020–2025, respectively), with a notable change-point occurring in 2015 (ruptures library; Truong et al., 2020). The quantification of neural-pathway terms depicted in Figure 6D ranked the limbic system (47.5%), sensorimotor network (27.9%), and attention network (15.1%), followed by the default-mode network (12.9%), as the most frequently mentioned pathways in the literature.

### 3.6 Analysis of journals and co-cited journals

Journal co-citation analysis was conducted with VOSviewer. Supplementary Table 8 lists the ten most productive journals, with NeuroImage (123 publications) and Pain (122 publications) far exceeding the others. Impact factor (IF) was used as the main metric of journal influence, reflecting citation frequency, while the Journal Citation Indicator (JCI) captured the impact of recently published work. The IF, JCI, and h-index values in Supplementary Table 8 correspond to the 2024 Journal Citation Reports.

Journal co-citation analysis identifies the journals that are most frequently co-cited in the corpus, which helps orient reading and manuscript submission. The co-citation heatmap is shown in Figure 7A. As summarized in Supplementary Table 9, the top three journals surpassed all others with more than 6,000 citations each. NeuroImage recorded the highest citation frequencies. NeuroImage focuses on advances in neuroimaging data acquisition, analytical techniques, and modeling strategies for structure–function and brain–behavior relationships.

**FIGURE 7:**
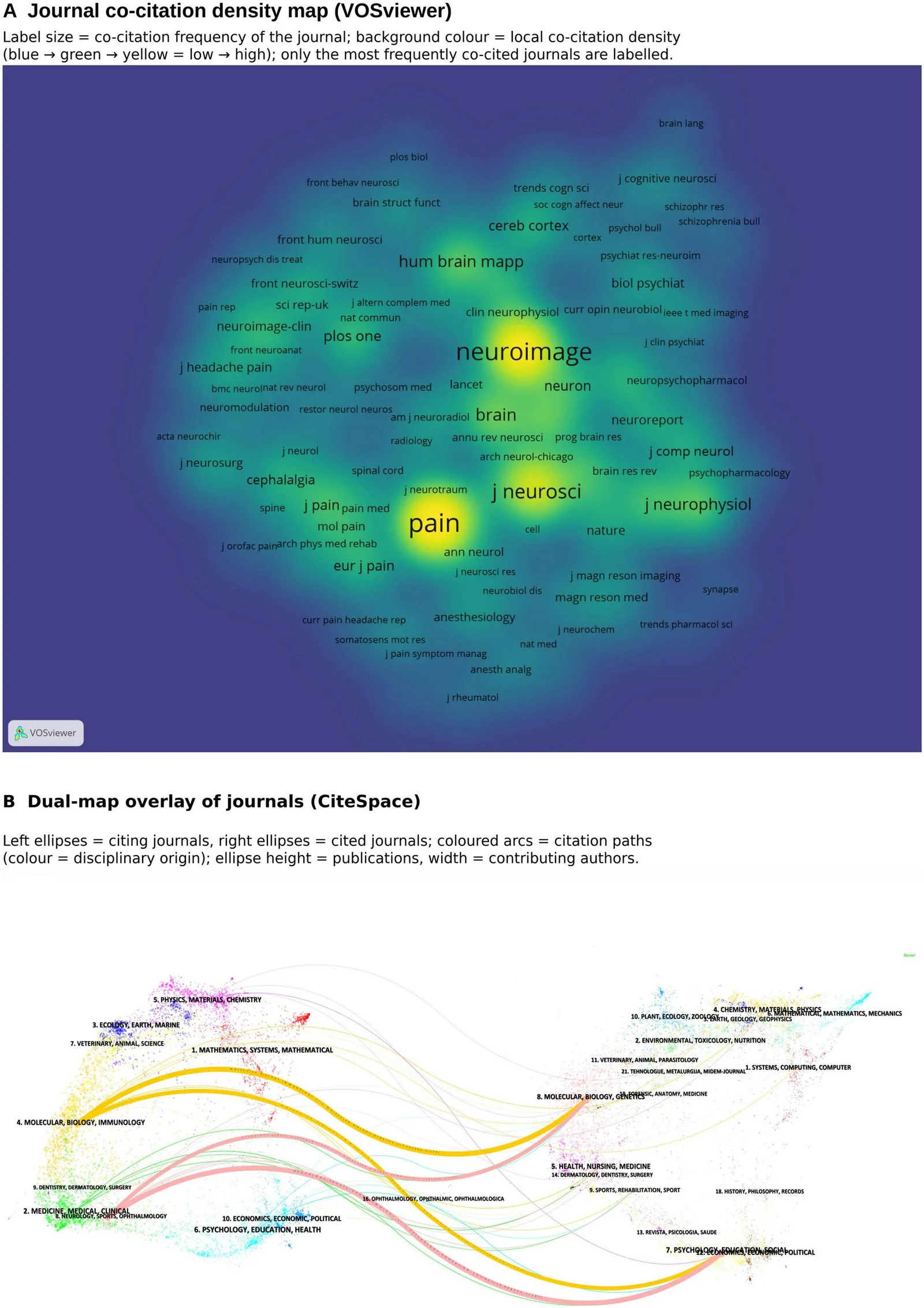
Analysis of journals and co-cited journals. **(A)** Co-citation analysis of the journals. **(B)** The dual-map overlay of journals. In panel **(A)**, label size denotes the co-citation frequency of the journal, background color denotes the local co-citation density (blue → green → yellow = low → high), and only the most frequently co-cited journals are labeled. In panel **(B)**, the left ellipses represent citing journals and the right ellipses cited journals; colored arcs denote citation paths, with colors indicating disciplinary origins; the vertical axis of each ellipse corresponds to publication counts and the horizontal axis to the number of contributing authors.

CiteSpace generated a dual-map overlay of journals (Figure 7B) to illustrate interdisciplinary integration and citation flows. The left map represents citing journals, and the right map shows cited journals. Citation paths begin with journals on the left and end with those on the right, with colors indicating disciplinary origins. In the overlay, the vertical axis of each ellipse corresponds to publication counts, while the horizontal axis reflects the number of contributing authors (Chen and Leydesdorff, 2013).

### 3.7 Analysis of co-cited references

CiteSpace reference clustering analysis (Figure 8A) produced a network of 914 nodes and 2,147 links, with a density of 0.0051, indicating relatively weak interconnections among references. As summarized in Supplementary Table 10, Apkarian et al. (2005) had the highest citation count (39) and burst strength (16.23), while Maihöfner et al. (2006) showed the strongest centrality (0.28) and Sigma index (6.23). Both studies ranked highest on these co-citation metrics. Apkarian et al. (2005) is a frequently cited cross-modal synthesis of the brain mechanisms of pain perception and modulation from the PET and early fMRI era that delineated the cortical and subcortical regions (S1, S2, insula, anterior cingulate and prefrontal cortices, and thalamus) subsequently examined in many fMRI studies, whereas Maihöfner et al. (2006) provided an early functional-imaging description of dynamic-mechanical allodynia in a neuropathic pain syndrome.

**FIGURE 8:**
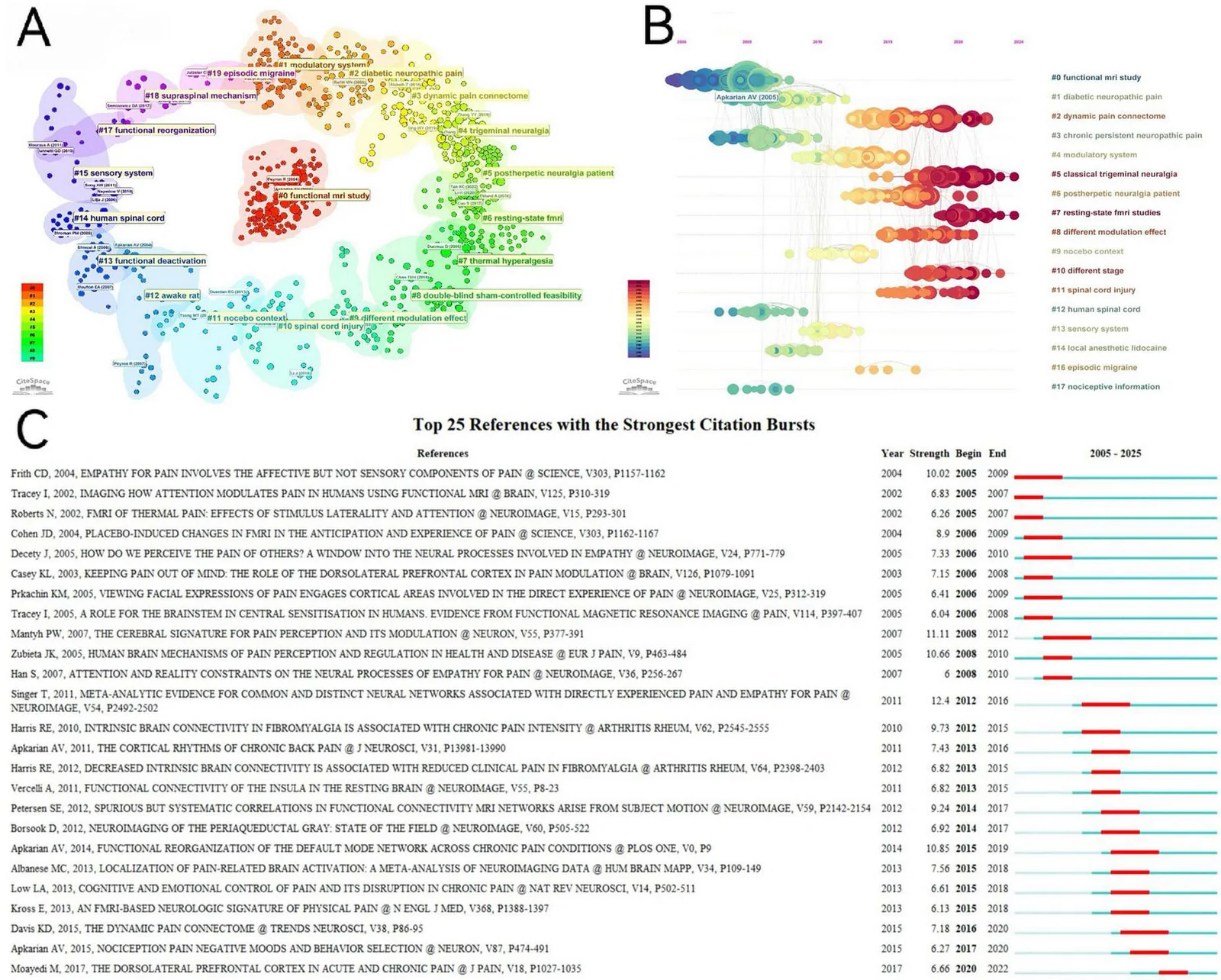
Analysis of co-cited references. **(A)** Map of reference clustering. **(B)** Cluster timeline plot of the co-cited references. **(C)** Top 25 references with the strongest citation bursts. In panels **(A,B)**, node size denotes citation frequency, node color denotes citation year (see the color bar in each panel), and links denote co-citation relationships; cluster labels (#0, #1,…) were generated with the log-likelihood ratio algorithm. In panel **(B)**, each horizontal band represents one cluster (legend at right), the horizontal node position denotes the year of first citation, and clusters are ordered top-to-bottom by size. In panel **(C)**, rows are sorted by burst onset year; horizontal bars span the analysis window 2005–2025, with light blue segments denoting years before the first citation, teal segments cited non-burst years, and red segments the burst interval (Begin–End); Strength is the Kleinberg burst intensity.

Clustering separates datasets into distinct groups by maximizing similarity within clusters and minimizing similarity between clusters. Post-clustering visualization displayed Modularity (Q = 0.8603) and Silhouette (S = 0.9385) values, which are key measures of clustering quality. Modularity assesses the significance of community structure within a network (range 0–1; Q > 0.3 indicates strong clustering). Silhouette evaluates cluster homogeneity, with values close to 1 reflecting high reliability (S > 0.7 indicating strong confidence). The values obtained in this analysis confirm excellent clustering validity.

Time-zone visualization of co-cited references (Figure 8B) illustrated temporal distributions and cluster densities. References within the same cluster aligned horizontally. The task-based fMRI cluster (#0) initially dominated with many large nodes but gradually shifted toward the resting-state fMRI cluster (#7), reflecting methodological maturation. The diabetic neuropathic pain cluster (#1) showed prolonged temporal activity, confirming its lasting research relevance. The dynamic pain connectomics cluster (#2) expanded into network-level investigations, advancing mechanistic understanding. Emerging clusters such as neuromodulatory effects (#8) highlighted growing interdisciplinary integration and clinical potential.

CiteSpace burst detection identified the 25 references with the strongest citation bursts, sorted by burst onset year (Figure 8C). In this analysis, “Year” marks the first year of citation, “Begin/End” define the duration of the burst, and “Strength” quantifies intensity. In the burst bars, light blue segments denote years before the first citation, teal segments denote cited non-burst years, and red segments denote the burst interval (Begin–End).

Citation dynamics showed two main phases: an early concentrated burst period (2002–2008) followed by a sustained progressive phase (2012–2020). Notably, research on prefrontal cortex mechanisms in pain processing (Ong et al., 2018) and pain modulation deficits in postherpetic neuralgia (Li et al., 2020) continued to generate citation bursts through 2025, as detailed in Figure 8C, Table 4, and Supplementary Table 10.

### 3.8 Co-occurrence analysis of keywords and keyword clustering

Figure 9A presents the keyword co-occurrence network generated by CiteSpace for the period 2005 to 2025. The network comprised 713 nodes and 3426 co-occurrence links, with a network density of 0.0135. The modularity Q value reached 0.8241, indicating a well-structured network with clear community divisions among the keywords. The weighted mean silhouette score was 0.9174, suggesting high homogeneity within clusters. Several keywords showed notably high co-occurrence frequencies. This analysis covers all keywords (author keywords plus index keywords); a complementary analysis restricted to author keywords after excluding core topic terms is reported later in this section. “pathophysiology” appeared 592 times, followed by “brain mapping” (573), “functional connectivity” (535), “nociception” (395), “brain region” (351), “chronic pain” (340), “insula” (339), “thalamus” (274), “brain function” (270), and “somatosensory cortex” (252). The network visualization revealed dense interconnections among terms related to neural structures, pain processing mechanisms, and functional imaging methodologies. Keywords such as “prefrontal cortex,” “amygdala,” “hippocampus,” “anterior cingulate,” and “cerebellum” occupied relatively prominent positions in the network topology. Additionally, terms addressing pain assessment and measurement, including “pain measurement,” “pain assessment,” “pain threshold,” and “hyperalgesia,” demonstrated substantial co-occurrence activity. The node colors ranging from dark blue to red reflected the temporal progression from 2005 to 2025, with more recently emerging keywords appearing in warmer tones. For readability, node labels in Figure 9A are shown for the top 1.0% of keywords by co-occurrence frequency.

**FIGURE 9:**
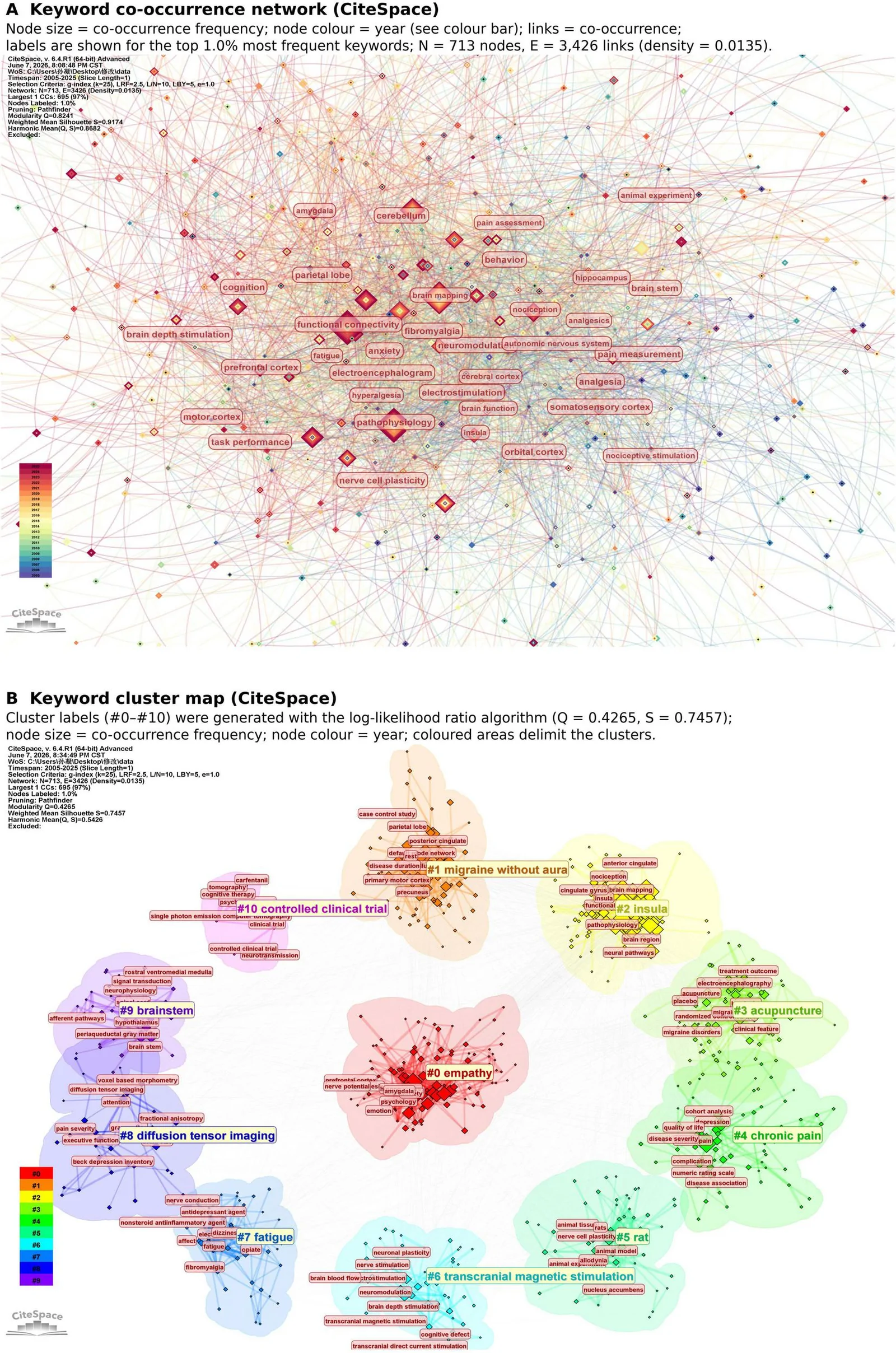
Co-occurrence analysis of keywords and keyword clustering. **(A)** Network visualization map of keywords co-occurrence analysis. **(B)** Map of keyword clustering. In panel **(A)**, node size denotes the co-occurrence frequency, node color denotes the year (dark blue = 2005 → red = 2025; see the color bar), and node labels are displayed for the top 1.0% of keywords by co-occurrence frequency (*N* = 713 nodes, E = 3,426 links, density = 0.0135). In panel **(B)**, cluster labels (#0–#10) were generated by the log-likelihood ratio algorithm (Q = 0.4265, S = 0.7457), node size denotes co-occurrence frequency, node color denotes year, and colored areas delimit the clusters.

Figure 9B displays the keyword clustering based on the co-occurrence network using the log-likelihood ratio algorithm, which identified 11 clusters (#0–#10) with a modularity of Q = 0.4265 and a weighted mean silhouette score of S = 0.7457, indicating reasonably good cluster separation and internal consistency. Three broad groupings emerged. Clusters centered on human brain regions and pain psychology comprised “empathy” (#0, 28 nodes, the largest), “insula” (#2, 19), “chronic pain” (#4, 16), “fatigue” (#7, 13), and “brainstem” (#9, 10); clusters centered on animal models and intervention or imaging methods comprised “rat” (#5, 15), “transcranial magnetic stimulation” (#6, 13), “diffusion tensor imaging” (#8, 11), and “controlled clinical trial” (#10, 10, the smallest together with #9); and the condition- and intervention-specific clusters “migraine without aura” (#1, 20) and “acupuncture” (#3, 17) lay between these poles. The spatial arrangement of clusters in the visualization accordingly showed some degree of separation between clusters focused on human brain regions and pain psychology and those centered on animal models and intervention methods.

Figure 10A provides an alternative temporal visualization of keyword emergence and evolution. To improve readability while retaining the full keyword set, node inclusion in the timeline overlay was controlled per 1-year time slice using the g-index with k = 2, yielding 72 nodes and 444 co-occurrence links (network density = 0.1737) after Pathfinder pruning. Keywords were arranged along a timeline from 2005 to 2025, with the horizontal position of each node indicating the year in which the keyword entered the selected network. Early-emerging keywords included foundational anatomical and imaging terms such as “prefrontal cortex,” “cingulate gyrus,” “somatosensory cortex,” “insula,” “nociception,” “pathophysiology,” “chronic pain,” and “magnetic resonance imaging.” The visualization showed dense keyword generation in the 2005 to 2010 period, with continued expansion through the subsequent years: “nerve network,” “cell network,” “brain function,” and “image processing” entered the network in 2007, “young adult” in 2009, and “physiology” and “thalamus” in 2010. Keywords entering the network in the middle phase included “bold signal” and “anterior cingulate” (2011), “visual analog scale” (2012), “functional neuroimaging” and “empathy” (2013), “procedures,” “psychology,” “connectome,” and “pathology” (2014), and “aged,” “anxiety,” and “blood” (2015). More recently appearing keywords included “diagnostic imaging,” “functional connectivity,” and “pain intensity” (2016), “rest” and “pain perception” (2017), “major clinical study” and “depression” (2019), “primary somatosensory cortex” (2020), and “questionnaire” (2022). The color coding of connections between keywords reflected temporal relationships, with warmer colors indicating more recent co-occurrence patterns.

**FIGURE 10:**
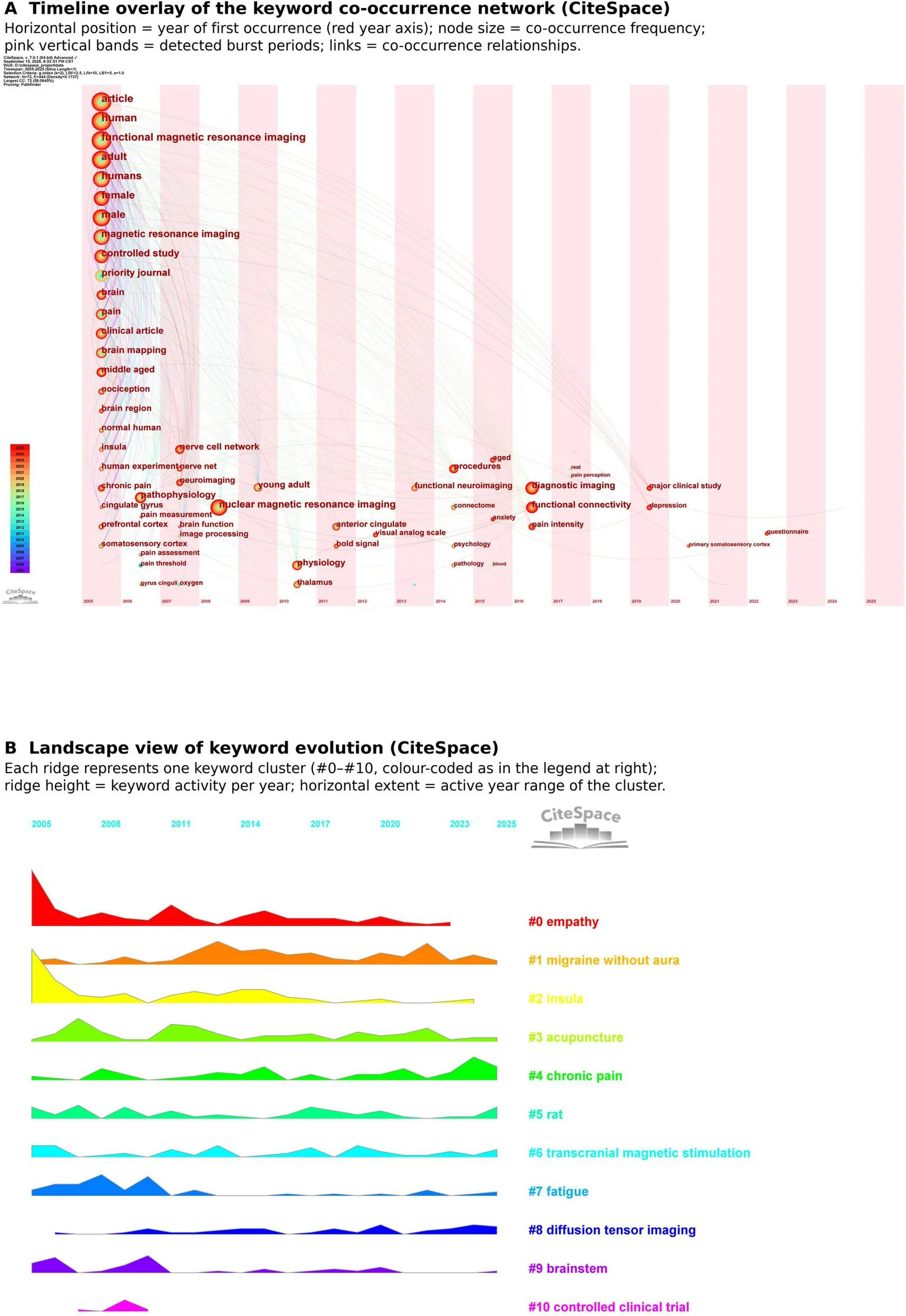
Temporal evolution of keywords. **(A)** Timeline overlay of the keyword co-occurrence network generated from the full keyword set in CiteSpace 7.0.1 (time slicing: 2005–2025, 1 year per slice; selection criteria: g-index with k = 2, LRF = 2.5, L/N = 10, LBY = 5, e = 1.0; pruning: Pathfinder; *N* = 72 nodes, E = 444 links, density = 0.1737; modularity Q = 0.1715, weighted mean silhouette S = 0.5146). **(B)** Landscape view of keyword evolution. In panel **(A)**, the horizontal position denotes the year of first occurrence (red year axis), node size denotes co-occurrence frequency, pink vertical bands denote detected burst periods, and links denote co-occurrence relationships. In panel **(B)**, each ridge represents one keyword cluster (#0–#10, color-coded as in the legend at right), ridge height denotes keyword activity per year, and the horizontal extent denotes the active year range of the cluster.

Figure 10B shows the timeline view of the 11 keyword clusters across the period from 2005 to 2025, with each horizontal band representing one cluster. Most clusters maintained continuous keyword activity throughout the study period and remained relevant through 2025, but their temporal profiles differed. Sustained, period-spanning activity characterized “empathy” (#0, active from 2005 onward with notable persistence into recent years), “insula” (#2, consistently present across multiple years), and “chronic pain” (#4, steady keyword generation with particular density in later years); by contrast, “migraine without aura” (#1) was concentrated in the middle years of the timeline, “transcranial magnetic stimulation” (#6) appeared more prominently in the latter half, and “diffusion tensor imaging” (#8) was confined to specific time windows, while “rat” (#5) and “fatigue” (#7) displayed intermittent or scattered yet persistent activity, the former reflecting ongoing animal-model research.

Table 5 lists the top 20 keywords with the strongest citation bursts detected by Kleinberg’s burst detection algorithm. The distribution of burst periods revealed that earlier bursts tended to center on anatomical brain regions and basic pain stimulation paradigms, while more recent bursts shifted toward methodological terms and clinical assessment instruments.

**TABLE 5:** Top 20 keywords with the strongest citation bursts (Kleinberg burst detection; sorted by burst onset year).

Rank	Keyword	Year	Strength	Burst period (Begin–End)
1	Amygdaloid nucleus	2005	27.61	2005–2015
2	Cingulate gyrus	2005	27.12	2005–2011
3	Somatosensory cortex	2005	25.97	2005–2012
4	Nociceptive stimulation	2005	16.71	2005–2012
5	Electric stimulation	2005	16.14	2005–2010
6	Functional laterality	2005	15.36	2005–2010
7	Evoked potentials	2005	14.09	2005–2011
8	Pain threshold	2006	20.12	2006–2012
9	Cerebral cortex	2005	12.12	2006–2011
10	Brain function	2005	11.97	2007–2013
11	Positron emission tomography	2007	11.97	2007–2013
12	Analysis of variance	2007	11.58	2007–2012
13	Nuclear magnetic resonance scanner	2013	34.56	2013–2017
14	Resting state network	2013	12.93	2014–2019
15	Nerve tract	2005	13.67	2015–2019
16	Cohort analysis	2015	13.01	2019–2025
17	Echo planar imaging	2006	18.84	2022–2025
18	Functional connectivity	2008	18.08	2022–2025
19	Quality of life	2012	13.07	2022–2025
20	Numeric rating scale	2020	11.54	2022–2025

To mitigate the influence of topic-defining search terms on the results, we conducted a keyword analysis on the author keywords after excluding three categories of terms: core terms associated with the research topic (neuropathic pain; fMRI/functional magnetic resonance imaging; brain), a curated list of generic non-informative words (e.g., treatment, response, control, effect), and bibliographic index descriptors (e.g., article, female, controlled study). Following this exclusion, 3,194 distinct author keywords remained across the 1,866 publications. As illustrated in Figures 11A, B and Table 2, the most prevalent keywords included “functional connectivity” (*n* = 214), “resting state” (*n* = 163), and “chronic pain” (*n* = 127). These were followed by “migraine” (*n* = 69), “insula” (*n* = 53), “default mode network” (*n* = 51), “empathy” (*n* = 45), “acupuncture” (*n* = 39), “fibromyalgia” (*n* = 39), “nociception” (*n* = 31), and “trigeminal neuralgia” (*n* = 31).

**FIGURE 11:**
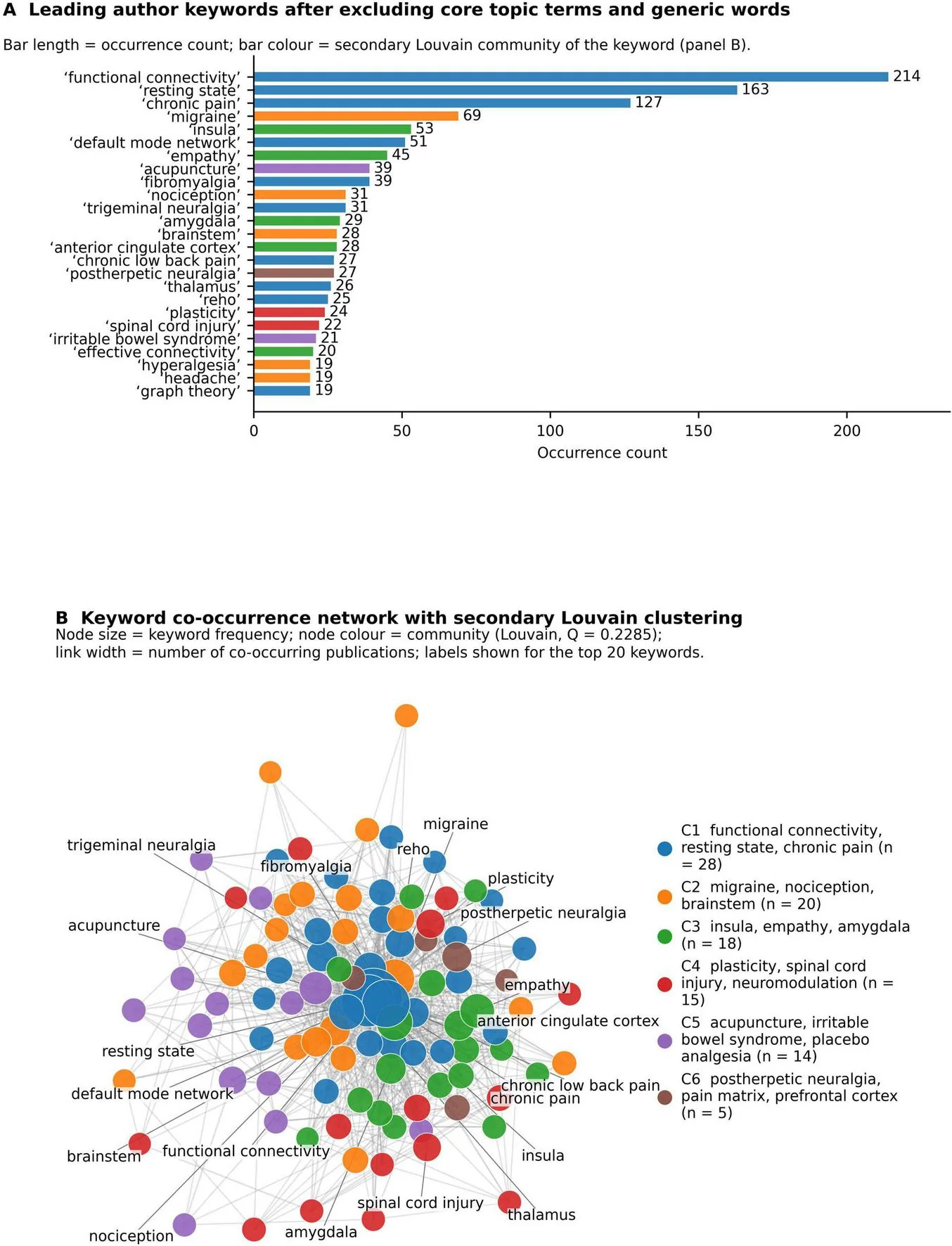
Author-keyword analysis after excluding core topic terms. **(A)** Leading author keywords after excluding the core topic terms (neuropathic pain, fMRI/functional magnetic resonance imaging, brain) and generic terms; bar length denotes occurrence count. **(B)** Keyword co-occurrence network of the 100 most frequent author keywords with secondary Louvain clustering (node size = keyword frequency, color = cluster). Link thickness denotes the number of co-occurrences between keywords; communities were detected with the Louvain algorithm (modularity Q = 0.2285). In panel **(A)**, bar color denotes the secondary Louvain community to which the keyword belongs in panel **(B)**. In panel **(B)**, node labels are shown for the top 20 most frequent keywords; the legend lists, for each community, the three leading terms by frequency and the community size.

Using the same exclusion criteria, a keyword co-occurrence network was constructed from the 100 most frequent author keywords, each appearing in five or more publications. The edges of this network were weighted according to the number of publications in which two keywords co-occurred, resulting in 100 nodes and 817 weighted edges (network density = 0.165). A subsequent community-detection analysis partitioned the network into six communities, whose leading members by keyword frequency are listed in Supplementary Table 13; the top three terms of each community were (1) “functional connectivity,” “resting state,” and “chronic pain” (28 keywords); (2) “migraine,” “nociception,” and “brainstem” (20 keywords); (3) “insula,” “empathy,” and “amygdala” (18 keywords); (4) “plasticity,” “spinal cord injury,” and “neuromodulation” (15 keywords); (5) “acupuncture,” “irritable bowel syndrome,” and “placebo analgesia” (14 keywords); and (6) “postherpetic neuralgia,” “pain matrix,” and “prefrontal cortex” (5 keywords). The keywords exhibiting the highest weighted betweenness centrality included “chronic pain” (0.078), “migraine” (0.059), “nociception” (0.054), “insula” (0.047), “resting state” (0.044), and “default mode network” (0.042), indicating that these terms frequently connected otherwise distinct groups of keywords.

Louvain community detection (Blondel et al., 2008; see Methods) was applied to the weighted keyword co-occurrence graph. Node bridging was assessed through weighted betweenness centrality. This partitioning resulted in six communities with a modularity value of Q = 0.2285. Since Q > 0.3 is typically regarded as indicative of a distinct community structure, this value indicates a modest modular organization, and the clusters should therefore be interpreted as indicative thematic groupings.

## 4 Discussion

### 4.1 Summary of main findings

This bibliometric analysis mapped 1,866 fMRI studies of neuropathic pain published between 2005 and 2025. The main findings are: (i) indexed annual output rose from 35 to 156 publications along a power-law trajectory, and the corpus has accumulated more than 94,000 citations; (ii) publication and citation leadership is concentrated in the United States and China, whereas bridging positions are held by several smaller producers; (iii) the thematic structure of the field has shifted from task-based activation paradigms toward resting-state functional connectivity, with growing use of machine-learning and multimodal imaging methods; and (iv) disease-specific connectivity patterns recur across the corpus but remain hypothesis-generating. These findings are discussed in detail below.

### 4.2 Research activity, productivity, and collaboration

The bibliometric indicators below describe the volume and distribution of research activity across the field. Annual output grew from 35 articles in 2005 to 156 in 2025, and the 1,866-article corpus accumulated 94,160 citations (mean 50.46 per article; corpus h-index 139). This sustained growth of the indexed literature indicates that fMRI has become an increasingly common tool in neuropathic pain research over the past two decades, although part of the increase may reflect expanding database coverage rather than field growth alone.

The United States (647 articles) and China (449) produced the largest volumes of literature, consistent with the size of their respective neuroscience and imaging research bases. In the country collaboration network, several comparatively small producers, including New Zealand, the Netherlands, and Belgium, occupied high betweenness-centrality positions. Because betweenness centrality measures how often a node lies on the shortest paths linking other nodes, it reflects a country’s bridging position within the collaboration network. High-centrality countries therefore occupy connective roles that can differ from their output or citation rankings, as the divergence between the productivity and centrality rankings in Supplementary Table 3 illustrates.

At the institutional level, output was led by Harvard Medical School (132 articles), Massachusetts General Hospital (110), and the University of Oxford (58), whereas the highest mean citation rates were observed for the University of Michigan and Northwestern University (Supplementary Table 4). Collaboration was concentrated within institutions and their affiliated hospitals and within national borders, indicating that broader cross-institutional and international partnerships remain comparatively limited.

The most prolific authors (Supplementary Table 5), including Napadow, Tracey, Kong, and Borsook, form a small number of established groups, and author co-citation (Supplementary Table 6) is led by Tracey, Davis, and Apkarian. As with centrality, productivity and co-citation counts quantify research activity and citation frequency within the corpus, identifying the field’s active groups and widely shared reference points.

### 4.3 Influential references and journals

The most co-cited references were Apkarian et al. (2005), a synthesis of pain-related brain mechanisms, and Maihöfner et al. (2006), an early functional-imaging study of allodynia in complex regional pain syndrome (Supplementary Table 10). Their prominence indicates that these works are probably common starting points for fMRI studies of neuropathic pain.

Pain and NeuroImage were the leading journals by both publication output and co-citation (Supplementary Tables 8, 9), consistent with the dual orientation of the field toward pain mechanisms and neuroimaging methodology. These journal-level metrics summarize where the literature is published and cited and are useful mainly for orienting submission and reading strategies.

The reference-burst and keyword-burst analyses trace how attention has shifted over time from early task-based activation paradigms and anatomical terms toward resting-state connectivity, computational methods, and clinical assessment instruments in more recent years (Figures 9, 10). These trends describe changes in research activity and vocabulary over time, characterizing how the field’s topics and methods have evolved.

### 4.4 Disease-specific fMRI findings across neuropathic pain conditions

The disease-level summaries in this subsection are narrative syntheses of representative primary studies cited in the corpus, rather than outputs of the bibliometric analysis itself. Conditions covered below were selected because they correspond to sustained clusters or themes in the corpus, for example, the task-based fMRI cluster in Figure 8B and the post-herpetic/regional theme in Figure 6A. Complex regional pain syndrome, phantom limb pain, and central post-stroke pain are underrepresented in the corpus and are therefore not covered. Where possible, the keywords and anatomical regions identified in the bibliometric analyses (for example, “thalamus,” “insula,” “default mode network,” and the limbic and sensorimotor pathways in Figures 6, 9, 10) are used below as anchors linking the quantitative results to the primary literature.

Cross condition comparison of the fMRI literature reveals both communalities and distinctions in brain network alterations across different neuropathic pain entities. A recent systematic review and meta-analysis of resting-state fMRI studies in chronic pain demonstrated decreased local efficiency in resting-state functional whole brain topology across chronic pain conditions, with impairments observed in both chronic primary and chronic secondary pain, suggesting that chronic pain is associated with similar reorganization of functional whole-brain topology (Butry et al., 2025). The default mode network (DMN) is a large-scale brain network comprising discrete, bilaterally symmetrical areas of the medial prefrontal, medial and lateral parietal, and medial and lateral temporal cortices, which decreases its activity during externally directed, attention-demanding tasks and is engaged during internally directed cognition (Raichle, 2015). This meta-analysis further confirmed that patients with chronic pain exhibit altered intra-DMN connectivity and increased connectivity between the DMN and somatosensory regions (Fiúza-Fernandes et al., 2025), supporting a shift toward global integration through stronger interconnectedness of self-referential processes and pain processing.

However, disease-specific patterns are increasingly recognized. In diabetic peripheral neuropathy (DPN), resting-state fMRI studies have demonstrated significantly reduced thalamocortical functional connectivity, particularly between the ventral posterior lateral and mediodorsal thalamic nuclei and the primary somatosensory cortex (S1), supporting the concept of thalamocortical dysrhythmia in chronic pain (Cauda et al., 2009; Croosu et al., 2022). Notably, a double dissociation has been reported: thalamus–insula functional connectivity was positively associated with neuropathic pain scores, while thalamus–somatosensory cortex connectivity was inversely correlated with the severity of peripheral nerve damage (Teh et al., 2021). These findings suggest that DPN is characterized by both sensory-discriminative disconnection and affective-processing hyperconnectivity.

In classic trigeminal neuralgia (CTN), dynamic functional connectivity analysis has revealed distinct temporal patterns characterized by complex coupling between the DMN and cognitive control network, with increased dwell time in a state showing tight DMN internal connections (Zhang et al., 2021). Patients with CTN also showed disrupted flexibility in state transition and global topological organization, with decreased temporal variability of efficiency in the anterior cingulate cortex (ACC) of the salience network and the thalamus and caudate nucleus of the subcortical network (Zhang et al., 2021). A more recent machine learning study further stratified trigeminal neuralgia subtypes using resting-state fMRI metrics, achieving 74.19% accuracy in discriminating TN patients from controls, with medial prefrontal cortex connectivity alterations serving as potential biomarkers (Wu et al., 2025).

Spinal cord injury (SCI)-related neuropathic pain demonstrates unique connectivity signatures. A systematic review of neuroimaging studies in SCI patients with neuropathic pain found primarily metabolite concentration alterations in sensory-pain processing regions, alongside bidirectional changes of brain structure (Huynh et al., 2020). Critically, a recent study differentiated neuropathic and nociceptive pain in chronic SCI using resting-state functional connectivity: intralimbic and limbostriatal connectivity alterations were uniquely associated with neuropathic pain severity, whereas thalamocortical and thalamolimbic connectivity alterations were specifically associated with nociceptive pain severity (Kowalski et al., 2023). This distinction underscores that neuropathic pain after SCI has a characteristic limbic-striatal signature that separates it from other pain types.

Postherpetic neuralgia (PHN) has been associated with disrupted functional connectivity between the amygdala and both the anterior cingulate cortex and prefrontal cortex, contributing to the anxiety–pain comorbidity frequently observed in PHN patients (Jiang et al., 2023). The basolateral amygdala–anterior cingulate cortex circuit has been specifically implicated in PHN-anxiety comorbidity, with altered causal connectivity in this pathway correlating with both pain intensity and anxiety scores (Jiang et al., 2025).

Collectively, these findings indicate that while all major neuropathic pain conditions share common alterations in the DMN, limbic system, and sensorimotor integration pathways, each condition exhibits distinct signatures in specific circuits: DPN shows thalamocortical disconnection, CTN shows DMN–cognitive control network dysregulation, SCI-NP shows limbostriatal reorganization, and PHN shows amygdala–prefrontal disconnection. These disease-specific patterns may carry translational implications, although each currently rests on a small number of studies; prospective validation is required before fMRI-based connectivity profiles can be considered as candidate biomarkers for differentiating NP subtypes, guiding mechanism-based treatment selection, or monitoring treatment response.

### 4.5 Thematic structure and its interpretation

Text-mining of the corpus summarizes the thematic structure of the published literature. The most prevalent themes were stimulus-evoked activation, brain-connectivity/default-mode, and post-herpetic/regional topics, and the limbic and sensorimotor systems were the most frequently mentioned neural pathways. These figures quantify how often topics and pathways are written about, indexing research activity and vocabulary preferences rather than establishing biological causality.

Read alongside the primary literature, these patterns are consistent with mechanistic studies that implicate prefrontal, insular, cingulate, and thalamic regions in the processing and modulation of neuropathic pain (Apkarian et al., 2005; Li et al., 2020; Ong et al., 2018). These primary studies provide the mechanistic evidence for the biological roles of these regions, and the present bibliometric data indicate that these themes dominate the published literature. The frequent co-mention of functional connectivity and the DMN is consistent with the broader shift in the neuroimaging field from task-based activation studies toward resting-state connectivity analyses, a transition that is well documented in the pain neuroimaging literature (Alshelh et al., 2018; Kucyi and Davis, 2015).

The corpus also shows growing use of machine-learning and multimodal-imaging methods, together with an increasing presence of connectivity-based and clinical-assessment terms in recent years. These observations identify emerging methodological directions within the literature. The translational value of these methods for the diagnosis and treatment of neuropathic pain will depend on the development of validated, reproducible imaging biomarkers that can be standardized across centers and integrated into clinical trial designs (Davis, 2019; Wager et al., 2013).

One additional thematic signal deserves comment. Acupuncture is a prominent term of the treatment/neuromodulation topic (Figure 6A), forms its own keyword cluster (#3 in Figure 9B), and peaked in author-keyword activity in 2025 (*n* = 39; Table 2), with a distinct keyword community linking “acupuncture,” “irritable bowel syndrome,” and “placebo analgesia” (Figure 11). This pattern indicates that acupuncture-related neuroimaging has become a visible and still-growing subdomain of the fMRI pain literature, frequently using imaging to probe the central mechanisms of needle stimulation and placebo responses. Because the present corpus was constructed around neuropathic pain terms, the position of acupuncture research within nociplastic and mixed pain conditions may be only partially captured here.

### 4.6 Resting-state versus task-based fMRI

The temporal evolution illustrated by our keyword-burst and reference-cluster analyses reveals a distinct methodological transition within the fMRI-NP field, shifting from an early predominance of task-based activation paradigms to an increasing emphasis on resting-state functional connectivity approaches. In the co-cited reference time-zone visualization (Figure 8B), the task-based fMRI cluster initially exhibited significant dominance with numerous large nodes; however, it gradually transitioned toward the resting-state fMRI cluster, indicating methodological maturation. This transition parallels broader advancements in neuroimaging techniques: resting-state fMRI captures spontaneous low-frequency fluctuations in the blood oxygen level–dependent (BOLD) signal without necessitating task performance, thereby rendering it particularly advantageous for investigating chronic pain populations that may struggle with structured experimental tasks (Bosma et al., 2018; Kucyi and Davis, 2015).

Evidence from both the bibliometric corpus and primary studies suggests that resting-state fMRI has yielded more consistent findings in chronic pain populations than task-based approaches. A systematic review and meta-analysis of resting-state fMRI in chronic pain found convergent evidence for altered intra-DMN connectivity, increased DMN–somatosensory connectivity, and decreased medial prefrontal cortex–periaqueductal gray connectivity across diverse chronic pain conditions (Fiúza-Fernandes et al., 2025). Similarly, graph-based meta-analysis found low-certainty evidence for decreased local efficiency in resting-state functional networks in chronic pain patients (Butry et al., 2025). In contrast, task-based fMRI studies have shown more variable results, likely due to heterogeneity in stimulus types, experimental designs, and analgesic interventions across studies.

However, several critical evidence gaps remain. First, direct comparisons between resting-state and task-based fMRI within the same NP patient cohorts are rare, making it difficult to determine whether one approach is more sensitive or specific for particular NP phenotypes. Second, the relationship between resting-state connectivity abnormalities and task-evoked activation changes within the same individuals is poorly understood. Third, longitudinal studies tracking the transition from acute to chronic neuropathic pain with both resting-state and task-based fMRI are scarce, limiting our understanding of how brain network alterations evolve over the natural history of the disease. Addressing these gaps will require prospective, multimodal imaging studies with standardized protocols that can be harmonized across centers.

### 4.7 From mechanism to clinical utility: biomarkers, prediction, and trial design

An important question facing the fMRI-NP field is whether neuroimaging findings can move beyond mechanistic description to serve as clinically useful tools for treatment prediction, patient stratification, and clinical trial endpoints. Our bibliometric analysis indicates that this translational trajectory is beginning: machine learning was mentioned in 3.1% of articles, and both the keyword-burst and reference-cluster analyses show increasing attention to connectivity-based methods and clinical-assessment instruments in recent years.

The development of the neurological pain signature (NPS) made a contribution to pain neuroimaging biomarker research (Wager et al., 2013). The NPS is a distributed brain pattern that predicts pain intensity across individuals and studies, showing high sensitivity and specificity (94% or more) for discriminating pain from non-painful warmth, pain anticipation, and pain recall. Importantly, the NPS response was suppressed by the opioid remifentanil, demonstrating pharmacological sensitivity. Subsequent work has extended this framework to predictive biomarkers: a support vector machine model using baseline resting-state functional connectivity patterns successfully distinguished responders to pregabalin from responders to milnacipran in fibromyalgia patients, achieving prediction accuracy significantly above chance (86% sensitivity, 77% specificity) (Davis, 2019). This study demonstrated that pretreatment brain connectivity could predict differential analgesic response to medications with distinct mechanisms of action. It should be noted that the NPS was developed and validated on experimental nociceptive pain; its specificity to neuropathic pain, and its ability to distinguish neuropathic from nociceptive pain, have not been established (Wager et al., 2013).

In the NP-specific literature, fMRI-based biomarkers are being developed for treatment prediction and patient stratification. A recent foundation-model-boosted multimodal learning framework (FMMTC) for fMRI-based neuropathic pain drug response prediction has demonstrated superior performance compared to unimodal baselines, with consistent cross-dataset adaptability (Fan et al., 2025). In trigeminal neuralgia, support vector machine models using resting-state fMRI metrics achieved 74.19% accuracy in discriminating patients from controls, suggesting potential for neuroimaging-guided patient stratification (Wu et al., 2025). These developments align with the broader vision of neuroimaging-based pain biomarkers and the U.S. National Institutes of Health (NIH) Helping to End Addiction Long-term (HEAL) Initiative, which has identified the development of objective pain biomarkers as a research priority.

For clinical trial design, fMRI biomarkers offer several potential applications: (1) enrichment biomarkers to select patients with specific neurobiological profiles likely to respond to targeted interventions; (2) surrogate endpoints to provide objective, mechanistically grounded measures of treatment effect; (3) patient stratification biomarkers to identify subgroups with distinct pathophysiological mechanisms; and (4) pharmacodynamic biomarkers to demonstrate target engagement at the neural systems level. However, before fMRI biomarkers can be integrated into clinical trials, several validation steps are required, including prospective replication in independent cohorts, standardization of imaging protocols and analysis pipelines, demonstration of test-retest reliability, and regulatory qualification pathways.

### 4.8 Methodological reproducibility challenges

The translation of fMRI findings from research to clinical applications is contingent upon methodological reproducibility, a challenge that has received insufficient attention in the NP neuroimaging literature. The Neuroimaging Analysis Replication and Prediction Study (NARPS), in which 70 independent teams analyzed the same fMRI dataset, found poor agreement in conclusions across teams, with methodological variability identified as the core source of divergent results (Botvinik-Nezer et al., 2020). This landmark study demonstrated that analytical flexibility in preprocessing pipelines, statistical models, and thresholding choices can produce markedly different results from the same raw data.

In the pain neuroimaging domain specifically, test-retest reliability studies have yielded mixed findings. A study examining the test-retest reliability of pain-related brain activity in healthy controls found that ACC and anterior insula responses to thermal pain showed generally “good” reliability over three consecutive scans, but with substantial inter-individual variability (Letzen et al., 2014). The reliability of functional connectivity measures appears more promising than that of task-evoked activation, with DMN connectivity showing moderate to good test-retest reliability across sessions (Meindl et al., 2010). However, reliability metrics remain unstandardized across studies, with some using intraclass correlation coefficients (ICC) of summary statistics and others calculating reproducibility ratios of voxel overlap, making cross-study comparisons difficult.

Several factors contribute to reproducibility challenges in fMRI-NP research. First, small sample sizes limit statistical power and increase the risk of false positives. Second, the heterogeneity of neuropathic pain conditions, comorbidities, and medication use introduces substantial between-subject variability. Third, preprocessing and analysis pipelines vary widely across laboratories, from motion correction and spatial normalization to connectivity estimation and statistical thresholding. Fourth, insufficient reporting of analytical details hampers replication efforts.

To address these challenges, the field should adopt emerging best practices: preregistration of study protocols and analysis plans to reduce analytical flexibility; multiverse analyses to explore the robustness of findings across different processing pipelines; standardized reporting frameworks such as the Committee on Best Practice in Data Analysis and Sharing (COBIDAS) checklist and open data and code sharing to enable independent replication. Collaborative, multi-center studies with harmonized imaging protocols and larger sample sizes will be essential for establishing robust, reproducible fMRI biomarkers in neuropathic pain.

### 4.9 Implications for future clinical trial design

The bibliometric patterns identified in this study, read alongside the primary literature cited above, suggest several hypothetical directions for the design of future clinical trials in neuropathic pain. We emphasize that the considerations in this subsection are perspective in nature: they are derived from bibliometric trends and narrative literature, not from experimental evidence generated by this study.

The disease-specific connectivity patterns summarized in section “4.3 Influential references and journals” raise the possibility that mechanism-based patient stratification could improve trial efficiency, for example by using connectivity phenotyping to define mechanistically more homogeneous cohorts in conditions such as painful diabetic neuropathy or spinal cord injury–related pain (Kowalski et al., 2023). These ideas remain hypothetical: the underlying connectivity signatures are based on small numbers of studies and have not been prospectively validated as enrichment biomarkers.

The growing citation bursts around connectivity-based methods and clinical-assessment instruments (Figures 9, 10) indicate that the field is moving toward composite endpoints that integrate neuroimaging measures with clinical outcomes. Such composite endpoints could provide more comprehensive assessment of treatment effects by capturing both symptomatic improvement and underlying neurobiological changes. However, the qualification of fMRI measures as drug development tools will require dedicated validation studies demonstrating that changes in fMRI metrics are clinically meaningful, responsive to treatment, and predict long-term patient outcomes. We also note that the shift toward resting-state methods may partly reflect their lower cost and logistical convenience rather than methodological superiority alone.

Against this background, and in an exploratory rather than prescriptive spirit, the following considerations may inform future trial discussions: (1) incorporating resting-state fMRI as an exploratory endpoint in early-phase trials to probe neural mechanisms of action; (2) exploring connectivity-based profiling for post-hoc responder analyses; (3) adopting standardized imaging protocols to enable cross-trial comparison; (4) prespecifying analysis pipelines and statistical thresholds to minimize analytical flexibility; and (5) encouraging collaboration between academic imaging centers and industry sponsors on biomarker validation. Whether fMRI measures can ultimately serve as qualified drug development tools will depend on dedicated prospective validation studies.

### 4.10 Study limitations

Although this study systematically charts the evolution of fMRI in neuropathic pain research using bibliometric and machine learning approaches, several limitations should be noted. First, reliance on the Web of Science Core Collection (SCI-EXPANDED), Scopus and PubMed ensured literature quality but excluded non-English publications and conference proceedings from databases such as Embase and CNKI. This may underrepresent contributions from non-English-speaking regions and introduce bias in collaboration analyses. Second, the exclusion of gray literature, including conference abstracts, may limit recognition of emerging technological trends. Third, the text-mining and regression analyses are descriptive summaries of the corpus: the random-forest model fitted to the annual publication series captured its shape with moderate accuracy (cross-validated R^2^ ≈ 0.70), and the topic- and keyword-level results depend on the chosen dictionaries and parameters. Future work could expand the corpora and dictionaries and apply more advanced text-analysis methods to characterize thematic structure more fully. In particular, the machine-learning analyses (topic modeling, embedding-based community detection, and exploratory link scoring) were used descriptively to summarize and visualize the corpus rather than to build validated predictive models; their outputs should be interpreted as exploratory, hypothesis-generating patterns, and alternative model parameterizations may yield different cluster solutions. Fourth, institution-level results depend on the parsing and harmonization of affiliation address strings; although departments and centers were attributed to parent organizations and name variants were merged using a documented thesaurus, residual misattribution cannot be fully excluded. Finally, as the field evolves rapidly, bibliometric analyses should be updated regularly to capture the most recent developments.

### 4.11 Conclusion

This study combined bibliometric analysis with machine learning methods to map two decades of fMRI research on neuropathic pain. Drawing on 1,866 articles published between 2005 and 2025, we found that annual indexed output rose from 35 to 156 publications along a power-law trajectory, and that the corpus has accumulated more than 94,000 citations. The United States and China lead in publication volume, but collaboration remains concentrated within national borders, while smaller producers such as New Zealand and the Netherlands hold key bridging positions in the network. Thematically, the field has moved from task-based activation paradigms toward resting-state functional connectivity, accompanied by growing use of machine-learning and multimodal imaging methods, with the limbic system, sensorimotor network, and DMN receiving the most attention. The disease-specific connectivity patterns identified in this corpus, including thalamocortical disconnection in diabetic peripheral neuropathy, default-mode dysregulation in trigeminal neuralgia, limbostriatal reorganization after spinal cord injury, and amygdala–prefrontal disruption in postherpetic neuralgia, may inform future, hypothesis-generating work on fMRI biomarkers for patient stratification and mechanism-based trial design, pending prospective validation. Realizing this potential will require larger multi-center cohorts, harmonized imaging protocols, preregistered analysis pipelines, and prospective biomarker validation. Given the pace of growth documented here, regular updates of this bibliometric mapping will be needed to keep the evidence base current.

## Data Availability

The raw data supporting the conclusions of this article will be made available by the authors, without undue reservation.
