# Supplementary material for: Functional magnetic resonance imaging in neuropathic pain: a bibliometric analysis incorporating machine learning
Source: Front Neurosci. 2026 Sep 30;20:1963495. doi: 10.3389/fnins.2026.1963495 (PMC13661907; doi:10.3389/fnins.2026.1963495)
Supplement: Supplementary file 1 [file Data_Sheet_1.pdf]

Supplementary Material

1 Supplementary Tables

Supplementary Table S1. Search strategies for Scopus and PubMed.

**Database**	**Search Strategies**
Scopus	TITLE-ABS-KEY(("neuralgia*" OR "neurodynia*" OR (neuropath* AND pain*) OR (neurogenic AND pain*) OR (neuralgi* AND pain*) OR (nerve AND pain*) OR "painful polyneuropath*" OR "painful radiculopath*")) AND TITLE-ABS-KEY(("fMRI" OR "functional magnetic resonance imaging" OR "BOLD-fMRI" OR "Functional MRIs" OR "functional MR imaging" OR "resting-state fMRI" OR "functional MRI" OR "rs-fMRI" OR "Resting State Functional Magnetic Resonance Imaging" OR "resting state fMRI"))
PubMed	(("neuralgia*"[Title/Abstract] OR "neurodynia*"[Title/Abstract] OR (neuropath*[Title/Abstract] AND pain*[Title/Abstract]) OR (neurogenic[Title/Abstract] AND pain*[Title/Abstract]) OR (neuralgi*[Title/Abstract] AND pain*[Title/Abstract]) OR (nerve[Title/Abstract] AND pain*[Title/Abstract]) OR "painful polyneuropath*"[Title/Abstract] OR "painful radiculopath*"[Title/Abstract])) AND ("fMRI"[Title/Abstract] OR "functional magnetic resonance imaging"[Title/Abstract] OR "BOLD-fMRI"[Title/Abstract] OR "Functional MRIs"[Title/Abstract] OR "functional MR imaging"[Title/Abstract] OR "resting-state fMRI"[Title/Abstract] OR "functional MRI"[Title/Abstract] OR "rs-fMRI"[Title/Abstract] OR "Resting State Functional Magnetic Resonance Imaging"[Title/Abstract] OR "resting state fMRI"[Title/Abstract])

Supplementary Table S2. Requirement package versions.

**Package name**	**Versions**
numpy	1.26.4
pandas	2.3.1
scikit-learn	1.7.1
scipy	1.13.1
gensim	4.3.3
umap-learn	0.5.9.post2
ruptures	1.1.9
networkx	3.4.2
torch	2.12.0+cpu
statsmodels	0.14.5
matplotlib	3.10.3
nltk	3.9.1

Supplementary Table S3. Ranking of countries in terms of centrality of publications.

**Rank**	**Centrality**	**Country**	**Total citations**	**Avg citations**	**h-index**
1	0.64	New Zealand	75	25.00	3
2	0.59	Netherlands	553	24.04	11
3	0.52	Germany	14261	66.33	59

4	0.51	China	10035	22.45	47
5	0.48	Belgium	1924	62.06	17
6	0.36	Australia	1739	54.34	17
7	0.31	United Kingdom	7335	67.92	47
8	0.30	Finland	352	117.33	3
9	0.29	Korea	1079	26.32	17
10	0.28	France	2904	74.46	20

Supplementary Table S4. Top 10 institutions by publications.

**Rank**	**Institution**	**Publications**	**Total citations**	**Average citations per article**	**h-index**
1	Harvard Medical School	132	10823	82.0	58
2	Massachusetts General Hospital	110	10956	99.6	58
3	University of Oxford	58	6104	105.2	36
4	University Medical Center Hamburg-Eppendorf	51	6403	125.5	33
5	Northwestern University	40	6529	163.2	32
6	University of Toronto	40	2689	67.2	24
7	University of Michigan	39	6929	177.7	32
8	University College London	39	3711	95.2	26
9	Peking University	37	2020	54.6	23
10	University of California, Los	35	2679	76.5	23

	Angeles				

Supplementary Table S5. Top 10 authors with the most publications.

**Rank**	**Author**	**Publications**	**Total citations**	**Average citations per article**	**h-index**
1	Napadow, Vitaly	41	4336	105.76	27
2	Tracey, Irene	31	3486	112.45	22
3	Kong, Jian	31	3123	100.74	28
4	Borsook, David	28	1874	66.93	18
5	Clauw, Daniel J.	24	3168	132.0	23
6	Harris, Richard E.	24	2602	108.42	22
7	Wager, Tor D.	24	2180	90.83	18
8	Apkarian, A. Vania	22	5021	228.23	15
9	Davis, Karen D.	22	1836	83.45	16
10	Becerra, Lino	22	1715	77.95	16

Supplementary Table S6. Top 10 co-cited authors.

**Rank**	**Co-cited author**	**Co-citations**	**Total citations**	**Average citations per article**	**h-index**
1	Tracey I	464	3526	106.85	23
2	Davis KD	354	1946	84.61	17
3	Apkarian AV	330	5175	178.45	18
4	Borsook D	289	2153	69.45	20
5	Raichle ME	256	487	162.33	3
6	Wager TD	254	2180	90.83	18
7	Garcia-Larrea L	253	23	7.67	1

8	Smith SM	243	445	63.57	6
9	Bushnell MC	237	2821	470.17	4
10	Büchel C	236	—	—	—

Supplementary Table S7. Thematic profile of high-frequency and co-cited authors. **Profiles were recomputed for the 12 most productive authors (by publication count) using document-level whole-word matching against the dictionaries in Supplementary Table S12; percentages denote the share of each author's papers matching a category, and only categories with a share ≥30% are listed.**

**Author**	Papers	**Research Directions**	**Research Methods**	**Research Conclusions**	**Neural Pathways**
Zhang, Y	53	Chronic Pain (38%)	fMRI (87%); FC (51%)	Pain-related Brain Regions (98%); Functional Connectivity Changes (66%); Treatment Response (49%); Biomarkers (49%); Gender Differences (38%); Structural Changes (34%)	Limbic System (53%)
Wang, Y	42	-	fMRI (81%); FC (50%)	Pain-related Brain Regions (88%); Functional Connectivity Changes (71%); Treatment Response (36%); Gender Differences (36%); Age-related (31%)	Limbic System (50%)
Napadow, V	42	Chronic Pain (43%)	fMRI (93%); FC (60%)	Pain-related Brain Regions (98%); Functional Connectivity Changes (74%); Treatment Response (71%); Gender Differences (38%)	Sensorimotor Network (48%); Limbic System (43%); Attention Network (36%); Default Mode Network (33%)
Liu, J	42	-	fMRI (86%); FC (57%)	Pain-related Brain Regions (90%); Functional Connectivity Changes (74%); Gender Differences (40%); Treatment Response (38%); Biomarkers (33%)	Limbic System (62%)

Zhang, J	40	-	fMRI (95%); FC (62%)	Pain-related Brain Regions (90%); Functional Connectivity Changes (78%); Treatment Response (38%); Biomarkers (38%); Gender Differences (32%); Age-related (32%)	Limbic System (55%)
Wang, X	36	-	fMRI (86%); FC (39%)	Pain-related Brain Regions (86%); Functional Connectivity Changes (61%); Treatment Response (58%); Biomarkers (31%)	Limbic System (53%)
Tracey, I	33	Pain Mechanisms (45%)	fMRI (91%); FC (30%)	Pain-related Brain Regions (100%); Functional Connectivity Changes (48%); Treatment Response (48%); Gender Differences (39%); Age-related (30%)	Limbic System (55%); Sensorimotor Network (39%)
Borsook, D	31	Chronic Pain (39%)	fMRI (90%)	Pain-related Brain Regions (100%); Functional Connectivity Changes (52%); Treatment Response (45%); Gender Differences (45%); Age-related (35%)	Limbic System (45%); Sensorimotor Network (32%)
Kong, J	31	Chronic Pain (32%); Pain Modulation (32%)	fMRI (87%); FC (68%)	Pain-related Brain Regions (100%); Functional Connectivity Changes (87%); Treatment Response (65%)	Limbic System (61%)
Apkarian, AV	29	Chronic Pain (90%); Neuroplasticity (31%)	fMRI (83%); FC (52%)	Pain-related Brain Regions (100%); Functional Connectivity Changes (76%); Treatment Response (41%); Gender Differences (31%)	Limbic System (55%)
Li, J	28	-	fMRI (89%); FC	Pain-related Brain Regions (96%); Functional	Limbic System (68%); Sensorimotor

			(71%)	Connectivity Changes (82%); Gender Differences (46%); Treatment Response (32%); Biomarkers (32%)	Network (36%)
Wang, L	28	-	fMRI (86%); FC (46%)	Pain-related Brain Regions (82%); Functional Connectivity Changes (64%); Treatment Response (32%)	Limbic System (46%)

Supplementary Table S8. Top 10 journals with the most output.

Rank	Output	Journals	IF	JCI	h-index
1	123	neuroimage	4.5	1.43	320
2	122	pain	5.5	1.93	238
3	61	plos one	2.6	0.85	268
4	60	human brain mapping	3.3	1.06	171
5	55	journal of neuroscience	4	1.24	422
6	30	journal of pain	4	1.12	109
7	28	frontiers in neurology	2.8	0.71	49
7	28	journal of headache and pain	7.9	1.97	50
9	27	journal of pain research	2.5	0.7	37
10	26	frontiers in neuroscience	3.2	0.77	71
10	26	scientific reports	3.9	1.07	149

Supplementary Table S9. Top 10 co-cited journals.

Rank	Citation	Journals	IF	JCI	h-index

1	11142	neuroimage	4.5	1.43	320
2	6788	journal of neuroscience	4	1.24	422
3	6437	pain	5.5	1.93	238
4	3527	proceedings of the national academy of sciences of the united states of america	9.1	2.31	699
5	3219	european journal of pain	3.4	1.09	96
6	2967	human brain mapping	3.3	1.06	171
7	2962	plos one	2.6	0.85	268
8	2718	brain	11.7	3.4	308
9	2240	cerebral cortex	2.9	0.85	223
10	1456	neuron	15.3	3.76	430

Supplementary Table S10. Top 10 co-cited references by frequency.

**Rank**	**Cited reference**	**Frequency**	**Centrality**	∑	**Burst**	**Half-life**
1	Apkarian AV (2005)	39	0.04	1.91	16.23	2.5
2	Ong WY (2019)	20	0.02	1.13	8.04	2.5
3	Peyron R (2004)	19	0.02	1.21	8.50	2.5
4	Maihofner C (2005)	17	0.03	1.24	7.00	2.5
4	Maihofner C (2006)	17	0.28	6.23	7.35	1.5
4	Schweinhardt P	17	0.02	1.14	7.17	1.5

	(2006)					
4	Cao S (2017)	17	0.01	1.09	7.20	2.5
4	Becerra L (2006)	17	0.06	1.55	7.17	2.5
10	Ducreux D (2006)	16	0.06	1.51	6.74	2.5
10	Zambreanu L (2005)	16	0.04	1.33	7.15	2.5
10	Maihofner C (2004)	16	0.02	1.18	7.36	2.5

Supplementary Table S11. Features used by the exploratory collaboration link-scoring model.

**Feature**	**Definition**
country1_publications	Total number of corpus publications of the first country of the pair
country2_publications	Total number of corpus publications of the second country of the pair
country1_fmri_focus	Proportion of the first country's publications whose keywords mention fMRI
country2_fmri_focus	Proportion of the second country's publications whose keywords mention fMRI
country1_clinical_focus	Proportion of the first country's publications whose keywords contain clinical terms (clinical, patient, or treatment)
country2_clinical_focus	Proportion of the second country's publications whose keywords contain clinical terms (clinical, patient, or treatment)
publication_diff	Absolute difference between the two countries' publication counts
focus_complementarity	Absolute difference between the two countries' fMRI-focus proportions
network_proximity	Adamic–Adar index of the country pair in the observed collaboration network
research_duration_diff	Absolute difference between the two countries' research-active spans (last minus first publication year)

Supplementary Table S12. Term lists of the predefined dictionaries used for text mining (whole-word matching on the concatenated title, abstract, author keywords, and index keywords of each record).

**Category**	**Terms (whole-word matching)**
Method: fMRI	fmri; functional magnetic resonance; bold; functional mri; functional mr imaging
Method: EEG	eeg; electroencephalography; electroencephalogram
Method: MEG	meg; magnetoencephalography
Method: PET	pet; positron emission tomography
Method: DTI	dti; diffusion tensor imaging; tractography
Method: VBM	vbm; voxel based morphometry; voxel-based morphometry
Method: Functional connectivity (FC)	functional connectivity; connectivity analysis; fc
Method: ICA	ica; independent component analysis
Method: GLM	glm; general linear model
Method: ROI	roi; region of interest
Method: MVPA	mvpa; multivariate pattern analysis
Method: Machine learning	machine learning; artificial intelligence; deep learning; random forest; support vector
Conclusion: Pain-related brain regions	pain; nociceptive; somatosensory; insula; anterior cingulate
Conclusion: Functional connectivity changes	functional connectivity; connectivity; network; default mode
Conclusion: Structural changes	structural; gray matter; white matter; volume; thickness
Conclusion: Treatment response	treatment; therapy; intervention; response; efficacy
Conclusion: Biomarkers	biomarker; marker; predictor; correlation
Conclusion: Gender differences	gender; sex; male; female; difference
Conclusion: Age-related	age; aging; elderly; young; adolescent

Pathway: Spinothalamic pathway	spinothalamic; anterolateral; lateral spinothalamic
Pathway: Corticospinal pathway	corticospinal; pyramidal; motor pathway
Pathway: Limbic system	limbic; amygdala; hippocampus; cingulate; insular; accumbens; olfactory; entorhinal
Pathway: Default mode network	default mode; dmn
Pathway: Sensorimotor network	sensorimotor network; somatosensory; motor cortex
Pathway: Attention network	attention; salience; executive
Pathway: Pain matrix	pain matrix; nociceptive matrix
Direction: Pain mechanisms	pain mechanism; nociception; nociceptive; central sensitization
Direction: Brain networks	brain network; functional network; connectome; network connectivity
Direction: Neuroplasticity	plasticity; reorganization; remodeling
Direction: Chronic pain	chronic pain; persistent pain
Direction: Acute pain	acute pain; evoked pain; experimental pain
Direction: Pain modulation	pain modulation; descending modulation; analgesia

Supplementary Table S13. Leading members of the six author-keyword communities detected by the Louvain algorithm on the co-occurrence network of the 100 most frequent author keywords (modularity Q = 0.2285).

**Community**	**Size**	**Leading members (by keyword frequency)**
1	28	functional connectivity; resting state; chronic pain; default mode network; fibromyalgia; trigeminal neuralgia; chronic low back pain; thalamus; reho; graph theory; independent component analysis; depression; voxel-based morphometry; brain networks; alff
2	20	migraine; nociception; brainstem; hyperalgesia; headache; allodynia; analgesia; spinal cord; central sensitization; functional imaging; connectivity; pain processing; hypothalamus; bold; cognition

3	18	insula; empathy; amygdala; anterior cingulate cortex; effective connectivity; emotion; dorsolateral prefrontal cortex; hippocampus; periaqueductal gray; pain modulation; anxiety; insular cortex; placebo; dynamic causal modeling; granger causality analysis
4	15	plasticity; spinal cord injury; neuromodulation; somatosensory cortex; neuroplasticity; stroke; spinal cord stimulation; phantom limb pain; transcranial magnetic stimulation; somatotopy; motor cortex; transcranial direct current stimulation; epilepsy; neuropathy; rat
5	14	acupuncture; irritable bowel syndrome; placebo analgesia; electroacupuncture; knee osteoarthritis; reward; brain imaging; low back pain; nucleus accumbens; randomized controlled trial; visceral pain; limbic system; dopamine; pathophysiology
6	5	postherpetic neuralgia; pain matrix; prefrontal cortex; prediction; herpes zoster

Supplementary Table S14. Affiliation harmonization thesaurus used for institution-level analyses.

Parsing rules: affiliation strings were parsed from the address (C1) field; the first comma-separated segment of each address line was taken as the candidate organization, and when it was a sub-unit (department, school, centre, or laboratory), the first subsequent segment denoting a top-level organization was used instead. Variants were harmonized as below, and each organization was credited once per record (full counting).

**Harmonized top-level organization**	**Address variants merged (case-insensitive, word-boundary, longest match first)**
Beth Israel Deaconess Medical Center	berenson-allen; beth israel deaconess
Boston Children's Hospital	boston children
Brigham and Women's Hospital	brigham and women
Charité – Universitätsmedizin Berlin	charité; charité universitätsmedizin; charite universitatsmedizin; charité universitaetsmedizin
Chinese Academy of Sciences	chinese academy of sciences

Fudan University	fudan university
Harvard Medical School	harvard medical school
Heidelberg University	central institute of mental health; heidelberg university; medical faculty mannheim; ruprecht karls; ruprecht-karls; university of heidelberg
Hospital for Sick Children	hospital for sick children; sickkids
Johns Hopkins University	johns hopkins
Maastricht University	university of maastricht
Mass General Brigham	mass general brigham
Massachusetts General Hospital	athinoula; martinos center; martinos center for biomedical imaging; massachusetts general; massachusetts general hospital; massachusetts general hospitals
McGill University	mcgill university
McLean Hospital	mclean and massachusetts general; mclean hospital
National Yang Ming Chiao Tung University	national yang ming university
Northwestern University	feinberg; northwestern university
Peking University	peking university
Queen's University	centre for neuroscience studies
Shanghai University of Traditional Chinese Medicine	shanghai university of traditional chinese medicine
Sichuan University	sichuan university; west china hospital
Spaulding Rehabilitation Hospital	spaulding rehabilitation
Stanford University	stanford university
Technical University of Munich	klinikum rechts der isar; technical university of munich; technische universität münchen; tu münchen
University College London	institute of child health; queen square; ucl; university college london; wellcome centre for human neuroimaging; wellcome department of imaging neuroscience; wellcome trust centre for neuroimaging

University Health Network	toronto western; university health network; toronto general hospital
University Hospital Essen	university hospital essen; universitätsklinikum essen
University Medical Center Hamburg-Eppendorf	university medical center hamburg; universitätsklinikum hamburg; university medical center hamburg-eppendorf; universitätsklinikum hamburg-eppendorf; universitaetsklinikum hamburg; eppendorf
University of Duisburg-Essen	university duisburg essen
University of Florida	university of florida
University of Michigan	chronic pain and fatigue research center; university of michigan
University of Oxford	john radcliffe; nuffield department; oxford university; university of oxford; wellcome centre for integrative neuroimaging
University of Toronto	university of toronto
Western Sydney University	university of western sydney

2 Supplementary Figures

Supplementary Figure S1. Machine-learning analysis of country collaboration. (A) Country collaboration communities (GraphSAGE + DBSCAN). (B) Collaboration networks across time windows. (C) Link-scoring probabilities for unlinked country pairs (random forest). In (A), node color denotes the GraphSAGE + DBSCAN community and node size denotes publication count. In (B), panels show the collaboration network within successive time windows. In (C), bars show the link-scoring probabilities of currently unlinked country pairs from the exploratory random-forest model (five-fold stratified cross-validated ROC-AUC = 0.921 ± 0.017); these scores are exploratory hypotheses, not predictions.
